# Integrating Porous Silicon Nanoneedles within Medical Devices for Nucleic Acid Nanoinjection

**DOI:** 10.1021/acsnano.4c00206

**Published:** 2024-05-10

**Authors:** Cong Wang, Chenlei Gu, Courtney Popp, Priya Vashisth, Salman Ahmad Mustfa, Davide Alessandro Martella, Chantelle Spiteri, Samuel McLennan, Ningjia Sun, Megan Riddle, Cindy R. Eide, Maddy Parsons, Jakub Tolar, John A. McGrath, Ciro Chiappini

**Affiliations:** † Centre for Craniofacial and Regenerative Biology, King’s College London, SE1 9RT London, U.K.; ‡ London Centre for Nanotechnology, King’s College London, WC2R 2LS London, U.K.; § Department of Pediatrics, Medical School, University of Minnesota, Minneapolis, Minnesota 55455, United States; ∥ Randall Centre for Cell and Molecular Biophysics, King’s College London, SE1 1UL London, U.K.; ⊥ Stem Cell Institute, University of Minnesota, Minneapolis, Minnesota 55455, United States; # St John’s Institute of Dermatology, King’s College London, SE1 7EP London, U.K.

**Keywords:** porous silicon, nanoneedles, topical delivery, advanced therapies, gene therapy, drug delivery system, medical devices

## Abstract

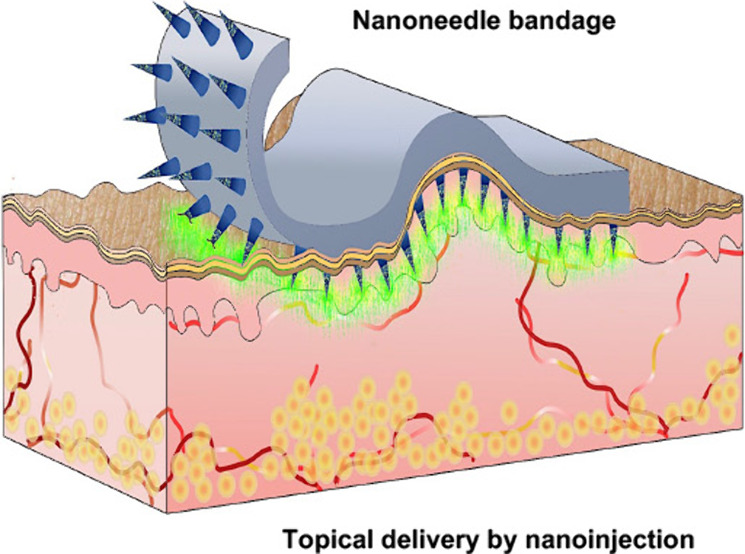

Porous silicon nanoneedles can interface with cells and tissues with minimal perturbation for high-throughput intracellular delivery and biosensing. Typically, nanoneedle devices are rigid, flat, and opaque, which limits their use for topical applications in the clinic. We have developed a robust, rapid, and precise substrate transfer approach to incorporate nanoneedles within diverse substrates of arbitrary composition, flexibility, curvature, transparency, and biodegradability. With this approach, we integrated nanoneedles on medically relevant elastomers, hydrogels, plastics, medical bandages, catheter tubes, and contact lenses. The integration retains the mechanical properties and transfection efficiency of the nanoneedles. Transparent devices enable the live monitoring of cell–nanoneedle interactions. Flexible devices interface with tissues for efficient, uniform, and sustained topical delivery of nucleic acids *ex vivo* and *in vivo*. The versatility of this approach highlights the opportunity to integrate nanoneedles within existing medical devices to develop advanced platforms for topical delivery and biosensing.

Arrays of vertical nanoprobes^1^ such as nanopillars,^2−4^ nanowires,^5−9^ nanostraws,^10,11^ nanotubes,^12,13^ and nanoneedles^14,15^ can manipulate and interrogate thousands of cells simultaneously by accessing the intracellular space with minimal perturbation of cell function. This ability is actively exploited to develop high-performing, minimally invasive platforms for biosampling,^16,17^ biosensing,^18,19^ and drug delivery^6,12,15,20^ in cell cultures and tissues. The scalable access to cells provided by nanoprobes through optimized cell–electrode interfaces can stimulate and record intracellular electrical activity in primary excitable cells, including neurons and cardiomyocytes.^21−25^ Among the many classes of vertical nanoprobes, porous silicon (pSi) nanoneedles^15,26^ are highly suitable for topical applications thanks to their *in vivo* biocompatibility and bioresorbability.^15,27^ Furthermore, pSi provides key advantages to nanoneedles, including high manufacturability with tunable nanoscale geometry^9^ (e.g., sharp needle-like tip) to minimize cell membrane disruption;^28^ a uniform and large surface area through tailored porosity^29^ to regulate drug loading efficiency^30^ and release kinetics;^31^ structural stability provided by the mechanical properties of silicon for stable interfacing with cells and tissue;^15^ bioresorbability;^32^ and high biocompatibility.^15,33^ Incorporating these nanoneedles in medical devices such as bandages or contact lenses would offer access to cells with minimal perturbation, providing better tools for minimally invasive topical therapeutics and diagnostics.^34^

However, porous silicon nanoneedles are conventionally manufactured on silicon wafers,^15,18^ which are rigid, fragile, and opaque, inevitably restricting their integration within medical devices. Hard, rigid substrates poorly match the mechanical properties of tissues, hampering natural tissue mobility while causing tissue damage and device failure.^35−37^ Moreover, their opacity prevents optical interrogation for monitoring and biosensing, limiting device functionality while restricting the possibility of tracking dynamic processes at the interface, including the real-time assessment of cell behavior and payload release. To tackle these limitations, several approaches have been developed to transfer silicon nanostructures to arbitrary substrates by mechanical detachment through razor blade peeling^38,39^ or controlled cracking.^40,41^ While simple, these methods have limited transfer rates, easily damage the nanostructures, are applicable to limited geometries, and require polymer encapsulation. Alternative methods include transfer printing,^42,43^ reactive ion etching,^44^ and replica molding.^45^ The recent integration of porosified, inverted needles on flexible substrates represented a significant step forward.^27^ For example, these needles could deliver chemotherapy for melanoma treatment or anti-inflammatory drugs for ocular treatment.^32,33^ Nevertheless, there is a need to extend the range of manufacturable morphologies for porous nanoneedles and the range of recipient substrates. Current transfer approaches still have limits on nanoneedle tip size, height, and aspect ratio, restricting the ability to manufacture short nanoneedles with sharp tips, which are the most efficient nanoprobes for the nonperturbing delivery of nucleic acids.^5,46^ Furthermore, porosification following needle formation limits the achievable porosity range and depth from the surface, restricting the control over bioresorption and payload release kinetics, which regulates biocompatibility and transfection efficiency.

Here, we propose a simple and robust way of integrating entirely and uniformly biodegradable pSi nanoneedles onto a wide range of medically relevant substrates, which include polydimethylsiloxane and poly(lactic acid) membranes, hydrogels, complex contours with concave structures, polypropylene catheter tubes, contact lenses, and medical bandages. We explored the synthesis parameters to tailor nanoneedle porosity, morphology, and geometry across the relevant range for nanoinjection. The resulting nanoneedle arrays across different geometries could be transferred to arbitrary substrates and handled with minimal loss of integrity. Transparent substrates enabled optical interrogation of nanoneedles interfacing with cells, revealing the evolution of the dynamic processes. The transferred nanoneedles retained their ability to transfect primary human cells with high efficiency. The nanoneedle-integrated devices could adapt without damage to the curvilinear, soft, elastic structure of tissues without suffering or procuring damage, enabling the uniform, efficient nanoinjection of nucleic acids into living organisms. These findings support the robustness of nanoneedle incorporation across a broad range of recipient substrates for nanoinjection, highlighting their potential for seamless integration within existing medical devices for implantation and topical delivery to the eye, skin, and mucosae.

## Results and Discussion

### Tailoring Porous Silicon Nanoneedles

**Figure 1:**
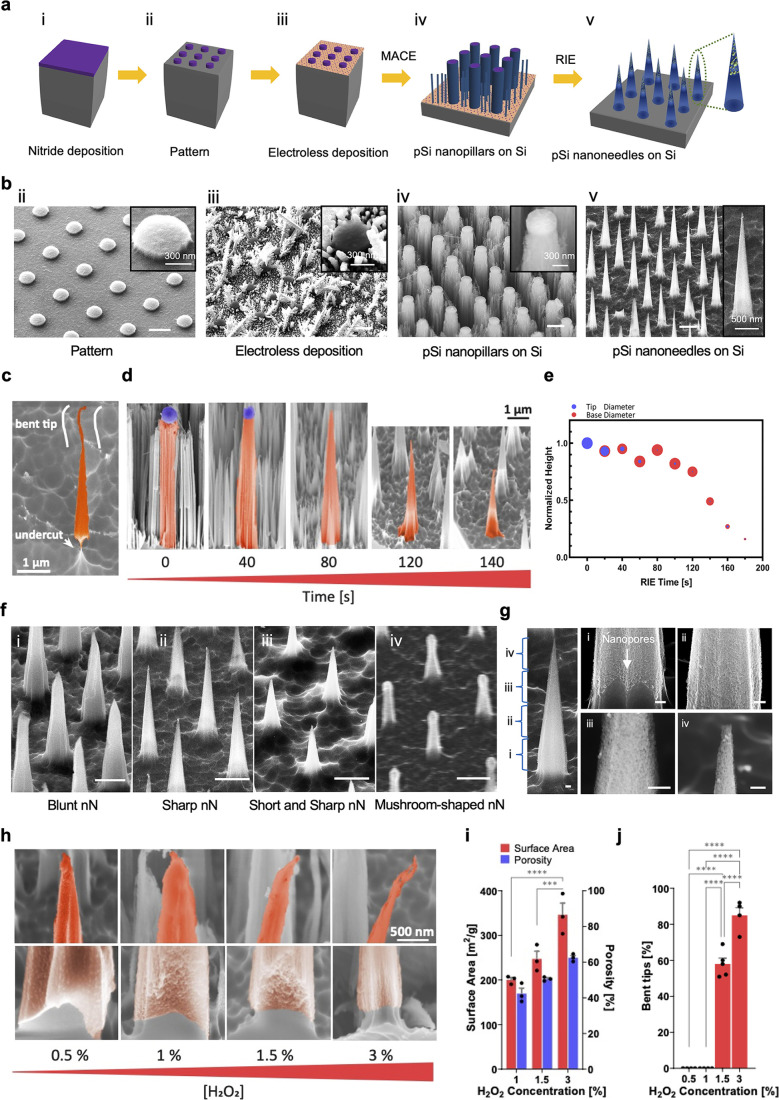
Tailoring porous silicon nanoneedles. (a) Schematic and (b) corresponding scanning electron microscopy (SEM) images of the pSi nanoneedles (nNs) fabrication process. (i) Deposition of low-stress nitride thin film by low-pressure chemical vapor deposition. (ii) Photolithographic patterning of silicon nitride in arrays of 2 μm spaced 600 nm diameter dots. (iii) Clusters of Ag nanoparticle deposition on silicon by electroless deposition. (iv) MACE formation of porous silicon nanopillars with distributed nanowires in between. (v) RIE in SF_6_ for shaping of nanocones and nanowire removal. Scale bars: 1 μm. (c) False-colored SEM image of a nanoneedle (nN) with bending tip and bottom undercut, indicators of low structural stability. (d) False-colored SEM images showing the RIE sharpening of nanoneedles between 0 and 80 s and shortening between 80 and 140 s. (e) Quantification of the RIE effect on nanoneedles as a function of time. Tip and base diameters are shown by the size of the blue and orange dots, respectively. Normalized height is shown by the *y*-axis position of the dot. (f) SEM images displaying the variety of obtainable nanoneedles. Scale bars: 1 μm. (g) SEM images showing the porous structure throughout the height of a nanoneedle at 1% H_2_O_2_ concentration. Scale bars: 100 nm. (h) False-colored SEM images of nanoneedles fabricated at different H_2_O_2_ concentrations. The bases of nanoneedles indicate only partial porosity for 0.5% [H_2_O_2_], while the tips show bending at 1.5% [H_2_O_2_] and increased tip splitting at 3%. Undercut is visible at 3% [H_2_O_2_]. (i) Brunauer–Emmett–Teller (BET) and density functional theory (DFT) fit of nitrogen sorption isotherms showing an increase in the surface area and porosity with the concentration of H_2_O_2_. *N* = 3 independent experiments. Two-way ANOVA with post hoc Tukey’s multiple comparisons test; ***p<0.001, ****p < 0.0001. (j) Quantification of tip bending as a function of H_2_O_2_ concentration. *N* = 4 independent experiments. One-way ANOVA with post hoc Tukey’s multiple comparisons test; ****p < 0.0001.

We started by exploring the range of achievable nanoneedle geometries with our established process in order to develop a library of nanostructures for integration with medical devices.^9,15,18^ The fabrication process (Figure 1a,b) comprised the deposition of a thin film of low-stress silicon nitride on a silicon wafer (Figure 1a-i), followed by the photolithographic patterning of a square array of 600 nm dots and its transfer into the silicon nitride by reactive ion etching (Figure 1a-ii,b-ii). A silver dendrite network, formed selectively on the exposed silicon surface by electroless deposition (Figure 1a-iii,b-iii), acted as the metal catalyst for the formation of an ordered array of porous nanopillars by metal-assisted chemical etching (MACE) (Figure 1a-iv,b-iv), which were then shaped into cones by reactive ion etching (Figure 1a-v,b-v) to form the nanoneedle array.

Reactive ion etching (RIE) is a key step to tailor the nanoneedle shape, which controls features such as the undercut and tip bending (Figure 1c) that yield low structural stability and may be detrimental to the interaction with tissues. Radio frequency (RF) forward power plays a key role in the sputtering vs etching balance of reactive ion etching.^47^ Increasing the RF power from 100 to 300 W (Figure S1) at constant pressure (100 mTorr) increased anisotropy, improving stability by hindering undercut and tip bending. The isotropy of lower power values made the nanoneedles thinner, uniformly along their heights, causing a loss of structural stability with a consequent high percentage of bent tips. Tip bending was present in 76% of nanoneedles at 100 W, extending to half of the nanoneedle height, and in 50% of nanoneedles at 200 W but was not present at 300 W. Undercut was similarly present only at 100 and 200 W (Figure S1). Increasing the RF power reduced the tip diameter and nanoneedle height without affecting the base diameter, supporting an increased etch rate (Figure S2). Reducing the base pressure could marginally restore anisotropy, producing shorter nanoneedles with sharper tips, less undercut, and less tip bending at 200 W (0% at 50 mTorr and 40% at 100 mTorr), while such a reduction did not have significant effects at 100 W (62% at 50 mTorr and 76% at 100 mTorr) and produced shorter and wider base nanoneedles at 300 W (Figure S3).

RIE shaped the nanoneedle in two stages (Figure 1d,e): first, it sharpened the tip while etching the protective silicon nitride layer between 0 and 80 s; then, it shortened the unprotected nanoneedle while preserving the conical shape with minimal further sharpening from 80 s onward. During the etching process, nanoneedles (Figure 1e) showed largely retained height (≥84%) and base diameter (≥90%) between 0 and 80 s. Tip diameter, in contrast, progressively decreased to 14% of the original size. Height and base diameter linearly decreased from 80 s onward until reaching 16% and 25% of their original dimensions at 180 s, respectively, while tip diameter remained constant (<20%). During shortening (80–180 s), height reduction was faster than base diameter reduction, thus preserving the conical shape while reducing the aspect ratio. RIE yielded a fine control over nanoneedle geometry. It was possible to manufacture structures of arbitrary height, ranging from nanopillars to nanomushrooms and nanocones with controllable tip sharpness and tip length (Figures 1f and S4). Undercut nanocones could serve to generate detachable nanoneedles for long-term implantation.

Metal-assisted chemical etching is also a key process in nanoneedle formation. The etching and porous characteristics of the nanostructures are controlled by the parameter , which can be tuned by modulating [H_2_O_2_] (Figure 1g,h).^48^ Scanning electron microscopy (SEM) and porosimetry indicated that increasing [H_2_O_2_] between 1% and 3% yielded nanoneedles that were porous from the tip through the base with increasing porosity and surface area (Figure 1h,i). Increasing [H_2_O_2_] beyond 1% reduced the mechanical stability of the resulting nanoneedles, as shown by the emergence of bent tips, base undercut, and tip splitting at 3% (Figure 1h). Using 0.5% [H_2_O_2_] formed solid silicon nanoneedles with limited roughening of the surface. Increasing H_2_O_2_ concentration increased the etching rate between 0.5% and 1.5%, while the erosion of nanoneedle tips emerging at 3% yielded a lower effective etching rate (Figure 1h,j). Increasing porosity with [H_2_O_2_] increased the RIE rate, yielding shorter nanoneedles at comparable RIE processing times. Metal-assisted chemical etching controlled the formation of solid and porous silicon nanoneedles, the details of their porous structure, and consequently their mechanical properties. The ability to regulate these parameters is essential to optimize the performance of vertical nanoprobes for cargo loading efficiency, its release kinetics, and the efficient interfacing with living systems.

### Substrate Transfer

**Figure 2:**
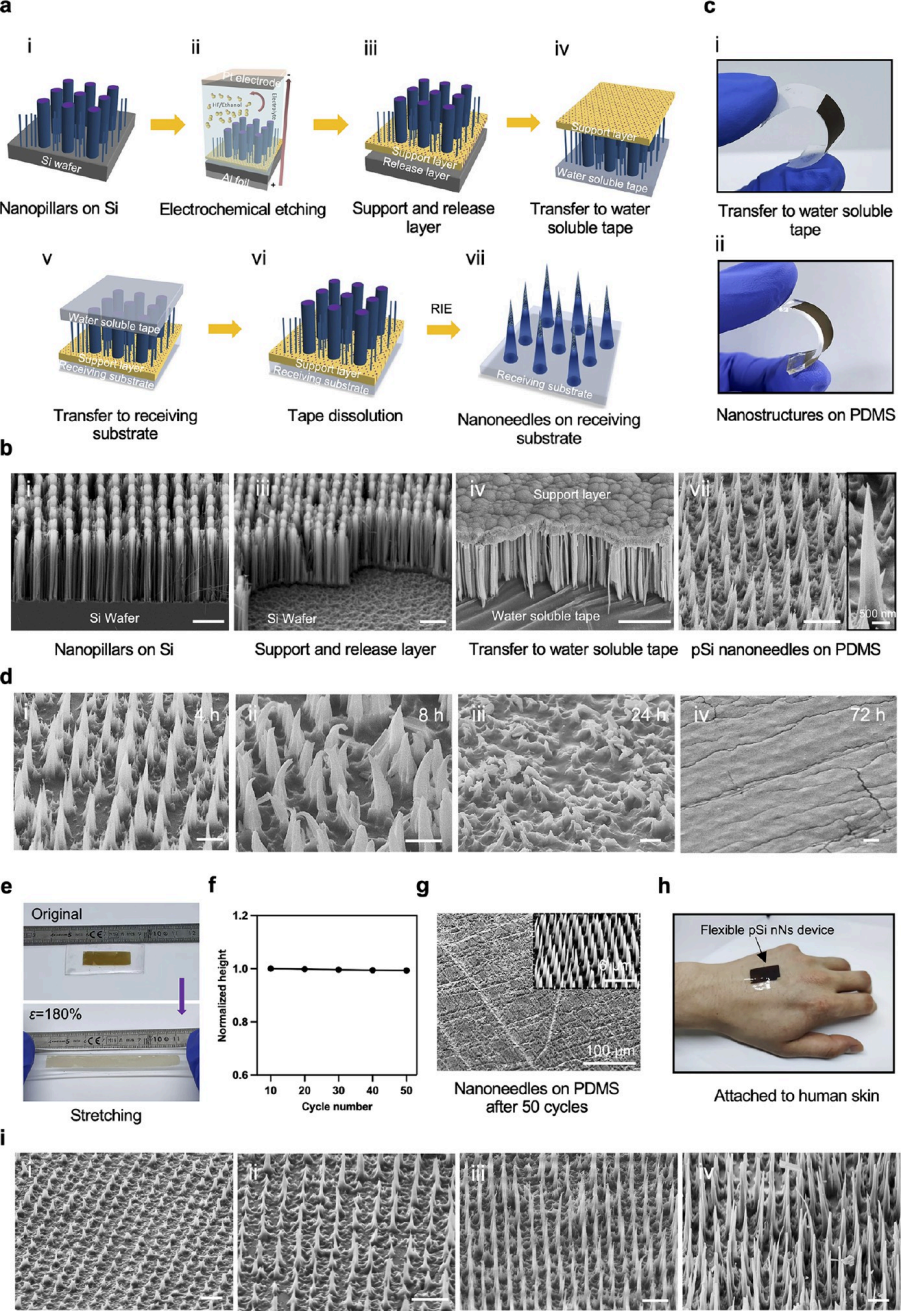
Nanoneedle substrate transfer. (a) Schematic illustration and (b) SEM images of transfer process. (i) Porous silicon pillars on Si substrate. (ii) Electrochemical etching (EC) to form (iii) support and release layers underneath the porous silicon pillars. (iv) Transfer to water-soluble tape. (v) Second transfer to final substrate. (vi) Tape dissolution. (vii) Reactive ion etching for nanoneedle shaping. Scale bars: 4 μm. (c) Images of the nanopillars on (i) water-soluble tape and (ii) polydimethylsiloxane (PDMS) elastomeric membrane. (d) SEM images of nanoneedles on PDMS incubated in cell culture medium at 37 °C. Nanoneedles display loss of structural integrity from 8 h and full dissolution at 72 h. Scale bars: 2 μm. (e) Images of pSi nanoneedles on PDMS during cyclic uniaxial stretching up to 180% strain. (f) Analysis of nanoneedle height during 50 cycles of stretching, normalized to initial height. (g) SEM image of nanoneedles on PDMS after 50 cycles. (h) Image of nanoneedles on thin PDMS membrane adhering to human skin while conforming to the complex three-dimensional contour of the tissue. (i) SEM images showing nanoneedles of different geometries integrated within a PDMS membrane. Scale bars: 4 μm.

We developed a versatile process to transfer centimeter-scale arrays of nanoneedles onto arbitrary substrates by combining MACE, RIE, and electrochemical etching. The process relies on generating a support layer with defined thickness (*T*_s_) over a release layer, both underneath the nanopillar array, by two-step electrochemical etching (Figures 2a,b and S5). The support layer of 600 nm thickness had low porosity, serving to maintain the arrangement and guarantee the mechanical integrity of the nanostructure array throughout the transfer process. The release layer had very high porosity, providing a loose connection to the original silicon substrate to facilitate handling while not opposing the uniform detachment of the nanostructure array and support layer during transfer. The electrochemical etching conditions could be tuned by modulating the solution concentration, applied current, and etching time to tailor the thickness and porosity of the two layers to optimize array stability and ease of detachment (Figure S5). The optimal support layer was obtained with 34 mA/cm^2^ for 60 s in a 1:3 HF/ethanol solution, while the optimal release layer was achieved with 101 mA/cm^2^ for 2 s in a 1:3 HF/ethanol solution.

The nanopillar array was removed from the silicon substrate using water-soluble adhesive tape, which was put in contact with the tips of the nanopillar array (Figure 2a-iv,b-iv). By simply peeling off the adhesive tape, the support layer, along with the nanopillar array, was easily separated from the donor Si wafer as a monolithic element over areas larger than 1 cm^2^ (Figure 2c-i and Movie S1). In the absence of the release layer, it was not possible to peel off the array using adhesive tape while maintaining its integrity (Movie S2). A 90° peel test confirmed that, in the presence of the release layer, detaching the nanoneedles from the Si wafer required a minimal force of less than 1*g* (Movie S3 and Figure S6). The detached array could then be transferred to a receiving substrate (e.g., polydimethylsiloxane, PDMS) by placing the water-soluble tape over it, with the support layer contacting the receiving substrate (Figures 2a-v and S7). We tested the application of pressure up to 500 kPa during the transfer process to assess whether it would affect nanoneedle integrity. When applying 10, 60, or 180 kPa (Figure S8), the nanoneedles remained intact, while the application of 500 kPa resulted in a significant loss of integrity. The tape was then dissolved, resulting in the transfer of the nanopillar arrays to the receiving substrate (Figures 2a-vi,c-ii and S9). This method ensured the retention of the orientation and spatial arrangement of the nanopillars over a large scale. The nanopillars could then be shaped into nanoneedles by RIE, which also regulated the thickness of the support layer (Figure 2a-vii,b-vii). Alternatively, RIE could be performed on the donor substrate, either before or after electrochemical etching, forming nanocones prior to transfer (Figure S10). The support layer provided mesoscale stability for the nanoneedle array on the receiving substrate while not affecting macroscopic flexibility. Its porosity could contribute to the loading capacity of the array while modulating the array dissolution and release kinetics in biological fluids.

The pSi nanoneedles and support layer were fully bioresorbable over the course of a few days (Figure 2d). Upon dissolution, nanoneedles retained a nanocone shape until 4 h with initial signs of loss of integrity from 8 h, and only the bases remained at 24 h until they were fully degraded at 72 h. The nanoneedles were flexible and stretchable (Figure 2e and Movies S4 and S5) without appreciable deviation from the properties of the PDMS substrate (20:1 ratio), where the elastic modulus could be controlled through the ratio of monomer to curing agent. When applying a uniaxial strain between 100% and 180%, the nanoneedles maintained their nominal height, shape, and arrangement (Figure S11) over the course of 50 cycles of uniaxial 180% strain (Figure 2f,g). Nanoneedle arrays on PDMS could deform elastically to match the complex three-dimensional contour of living tissues (Figure 2h). The versatility of this process enabled the integration of nanoneedle arrays with a broad range of geometries on the receiving substrate (Figure 2i).

### Device Integration

**Figure 3:**
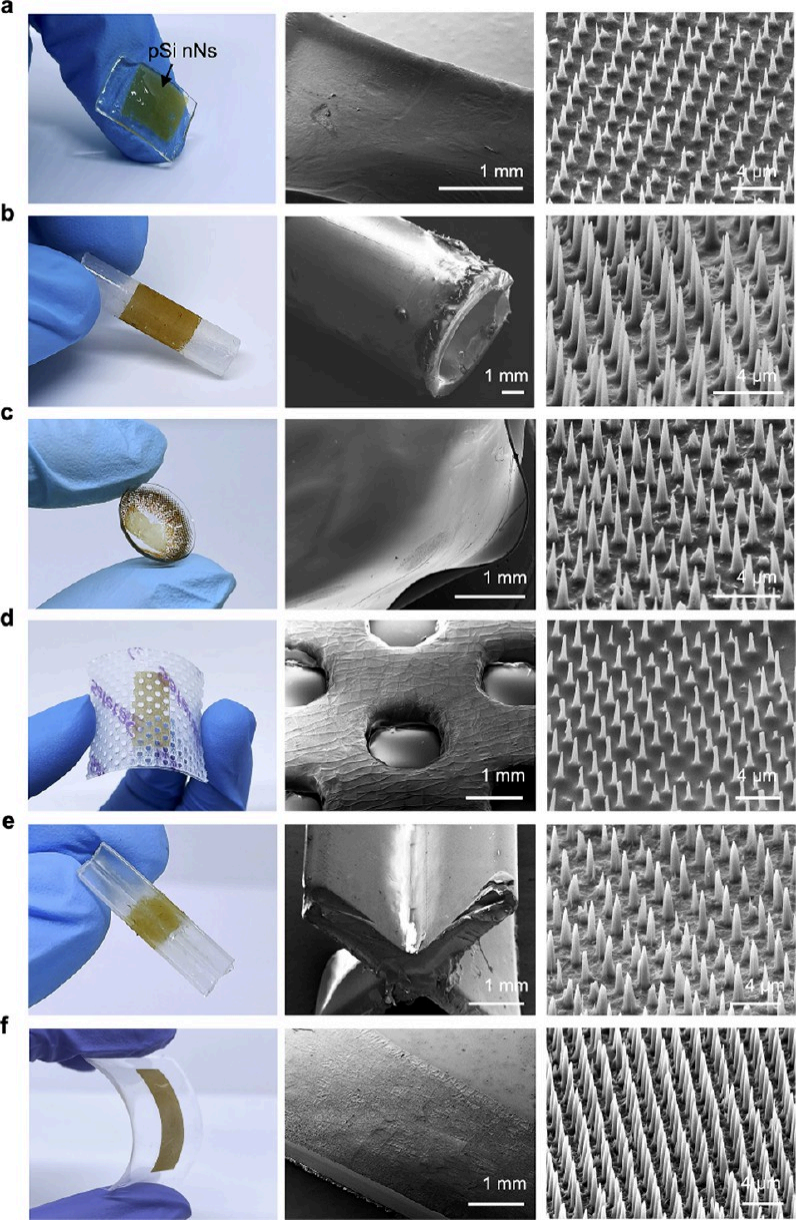
Device integration. Optical (left) and SEM (middle and right) images of pSi nanoneedles integrated within a (a) biodegradable gelatin hydrogel, (b) polypropylene catheter tube, (c) contact lens (ENVIE), (d) wound bandage (Mepilex, Mölnlycke), (e) sharp, convex structure, and (f) poly(lactic acid) film. The low-magnification SEM images show nanoneedles adhering uniformly throughout the surface, while the high-magnification SEM images show the integrity of the nanoneedle arrays.

To demonstrate the versatility of the transfer approach, we tested the integration of nanoneedles within devices with differing compositions, mechanical properties, shapes, and medical uses. The nanoneedles could be transferred onto biodegradable gelatin hydrogels (Figures 3a and S12a), which are suitable substrates for tissue engineering and biomedical applications that require full resorption and tissue-matching mechanical properties. Curved substrates such as polypropylene catheter tubes (Figure 3b) and contact lenses (Figures 3c and S12b) are well suited for developing medical devices for topical delivery; the pSi nanoneedle arrays conformed well to the curved surfaces of these materials. Nanoneedles integrated effectively with a wide range of bandages used for wound healing in the skin and mucosae (Figures 3d and S13). On sharp, convex plastic structures (Figure 3e) and transparent, plastic (poly(lactic acid)) substrates (Figure 3f), they integrated using a thin intermediate polymer layer as an adhesive. In all instances, the fundamental mechanism of this transfer approach could be applied to any nanotopography attainable on porous silicon layers and enabled transfer onto substrates across a broad range of physical and chemical properties to match the intended application.

### Live High-Content Imaging on Transparent Nanoneedles

**Figure 4:**
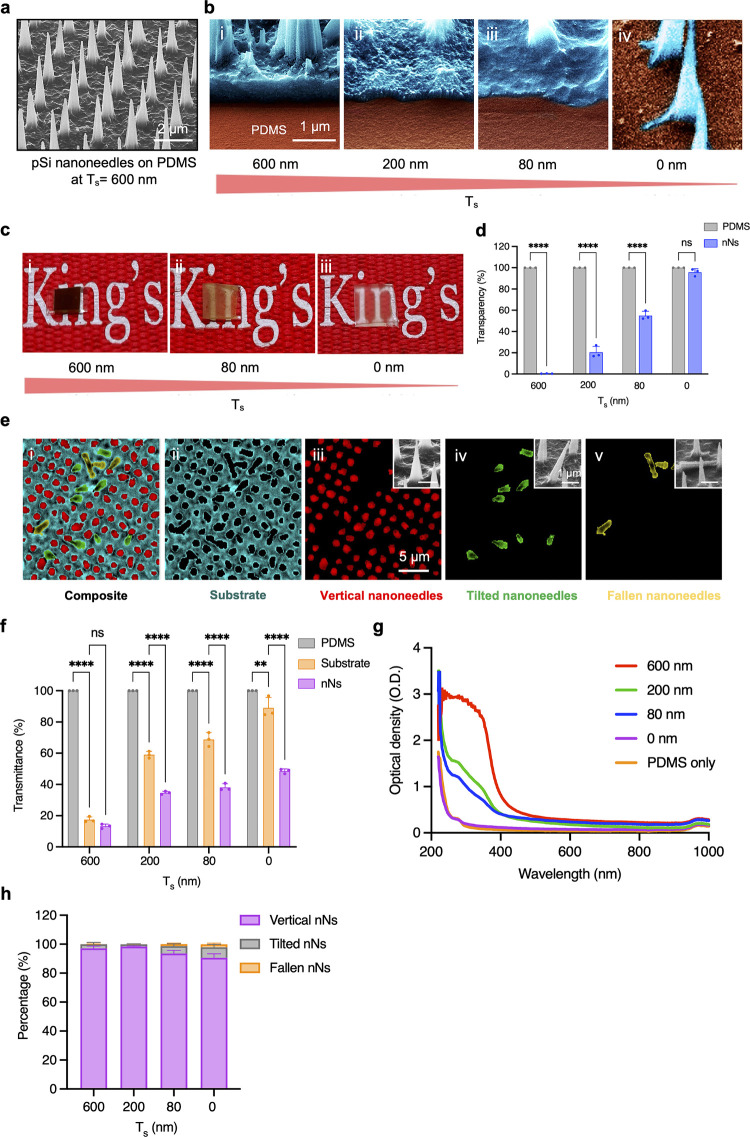
Tunable transparency. (a) pSi nanoneedles on PDMS at *T*_s_ = 600 nm. (b) False-colored SEM images of the interface between nanoneedles and PDMS as a function of *T*_s_ reduction by RIE: (i) 600, (ii) 200, (iii) 80, and (iv) 0 nm. Porous silicon in blue, PDMS in orange. (c) Optical images of the tunable transparency achievable by decreasing *T*_s_. A 600 nm *T*_s_ generates largely opaque nanoneedles, while reduction to 0 nm generates a transparent device. (d) Quantification of optical transparency as a function of *T*_s_. *N* = 3 independent experiments. One-way ANOVA with post hoc Tukey’s multiple comparisons test; ****p < 0.0001. (e) Segmentation of (i) a bright field image of nanoneedles on PDMS and classification of regions as (ii) substrate, (iii) nanoneedles retaining a vertical orientation, (iv) nanoneedles tilted from the vertical, and (v) fallen nanoneedles. (f) Quantification of transmittance for the PDMS substrate region and the nanoneedle region as a function of *T*_s_. Transmittance calculated from segmented images for substrate and nanoneedles. PDMS refers to the adjacent region of PDMS not covered by nanoneedles. *N* = 3 independent experiments. One-way ANOVA with post hoc Tukey’s multiple comparisons test; ****p < 0.0001. (g) Optical density as a function of wavelength over the UV–vis range for nanoneedles integrated with PDMS at different values of *T*_s_ = 600, 200, 80, and 0 nm. (h) Analysis of nanoneedle integrity as a function *T*_s_, calculated from image segmentation.

Through RIE, it was possible to control the transparency of the integrated device by tuning the thickness of the support layer (Figure 4a,b). Progressively reducing its thickness from the original 600 nm (Figure 4b-i) to 0 nm (Figure 4b-iv) increased the optical transparency of the device (Figure 4c,d) from 0% to 96%. The thinning process did not affect the nanoneedles, which retained their original morphology (Figure S14). Segmentation of a bright field microscopy image (Figure 4e-i) enabled the identification of the substrate (Figure 4e-ii) and nanoneedles (Figure 4e-iii–e-v). The analysis of the segmented images revealed separate contributions to transmittance from the nanoneedles, rising from 13% to 48% with decreasing *T*_s_, and from the support layer, raising from 17% to 89% (Figure 4f). Analysis of the ultraviolet–visible (UV–vis) spectra confirmed the increase in transmittance with decreasing *T*_s_ (Figure 4g)*.* The 600 nm *T*_s_ nanoneedles exhibited an elevated optical density, particularly for wavelengths below 400 nm. This optical density progressively decreased until the 0 nm *T*_s_ nanoneedles presented a UV–vis spectrum analogous to that of a planar PDMS layer. These data demonstrate that our transferred nanoneedles can offer optical transparency comparable to PDMS, providing a valuable tool for optical microscopy analysis of the cell–nanoneedle interface.

Reducing the support layer largely preserved the structural stability of the nanoneedles (Figure 4e,h) which is critical to their transfection efficiency. While lower thickness increased the fraction of nonvertical nanoneedles, in all conditions, the vast majority (>90%) remained vertical following transfer (Figure 4e-iii,h), with less than 10% tilted (1–7%) or fallen (0–3%) (Figure 4e-iv,e-v,h).

**Figure 5:**
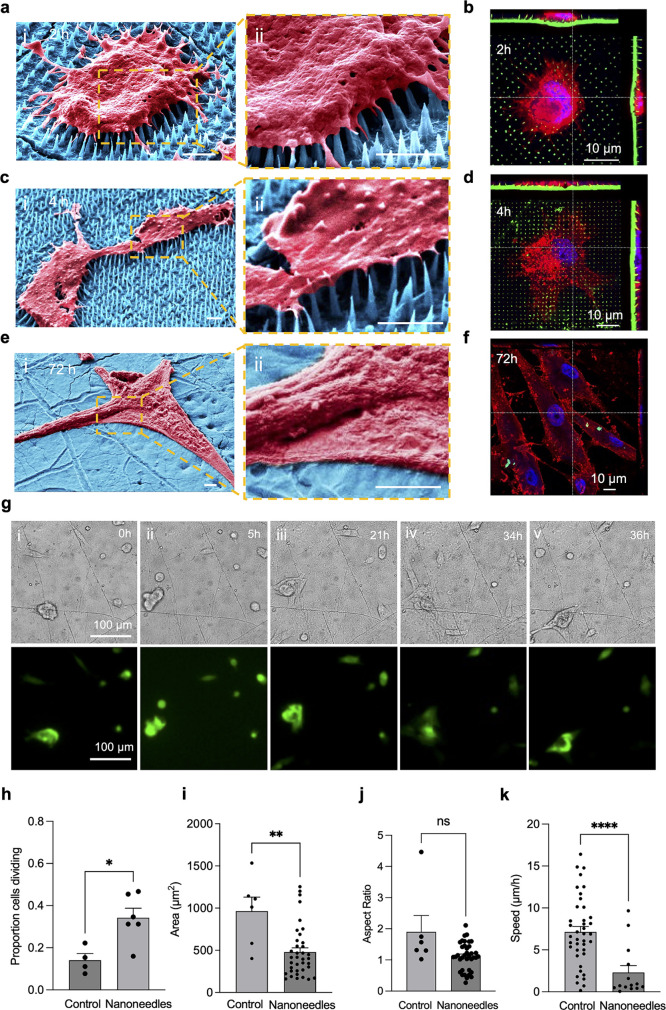
Cell interfacing and live high-content imaging. (a–f) False-colored SEM and confocal images of primary human dermal fibroblasts (hDFs) interfacing with nanoneedles at (a, b) 2 h after seeding; (c, d) 4 h after seeding, where cells extend protrusions and spread; and (e, f) 72 h after seeding, where nanoneedles are fully degraded. Scale bars: 4 μm. (g) Live high-content imaging of hDFs over 36 h in bright field and fluorescence. (h–k) Cell morphometric analysis from live high-content imaging on transparent nanoneedle substrates, indicating the (h) proportion of dividing cells, Mann–Whitney test, *p < 0.05; (i) cell area, Mann–Whitney test, **p < 0.01; (j) aspect ratio; and (k) cell velocity, Mann–Whitney test, ****p < 0.0001.

These transparent devices enabled investigating cell–nanoneedle interfacing and monitoring their interaction by live imaging. At 2 h after seeding, the tips of the nanoneedles appeared to interface with the cytosol, while cells retained a spherical shape, characteristic of the early stages of spreading (Figure 5a,b). As time progressed, cells adhered and spread, wrapping the tips of their filopodia around neighboring needles, which largely retained their integrity and vertical alignment (Figure 5c,d). Throughout the process of interfacing, the nanoneedles experienced progressive degradation, and at 72 h, the nanoneedles were fully degraded, while the cells adhered to the substrate (Figure 5e,f). Throughout the interfacing process, nanoneedle bending due to cellular forces was comparable to what was observed on silicon substrates, further supporting the mechanical stability of the transferred nanoneedles, even in the absence of the support layer (Figure S15).

Live high-content imaging of human dermal fibroblasts (hDFs) cultured on nanoneedles over PDMS and on a flat PDMS surface as a control revealed the dynamics of cell–nanoneedle interaction (Movies S6–S9). We could observe the evolution of the biointerface, monitoring cells progressively spreading and dividing over the nanoneedles (Figure 5g) and quantifying the proportion of dividing cells, cell area, aspect ratio, and speed. The number of cells that experienced division on the nanoneedles was notably higher than that on the control (Figure 5h). Cell area was reduced on the nanoneedles (Figure 5i), whereas the aspect ratio remained unchanged (Figure 5j). Furthermore, the cell velocity significantly decreased on the nanoneedles (Figure 5k).

### Cell Nanoinjection

**Figure 6:**
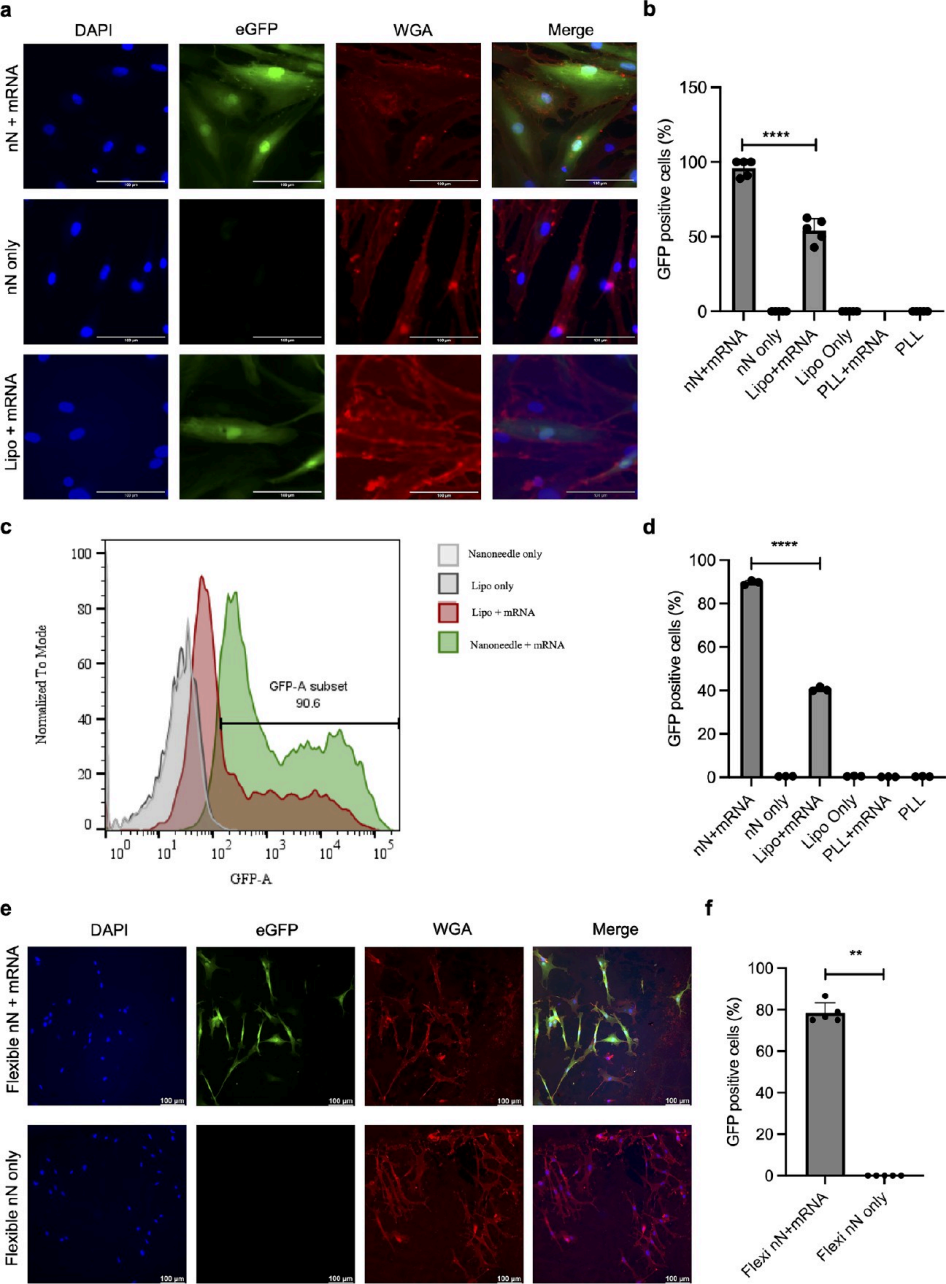
Nanoinjection of GFP mRNA. (a) Fluorescence microscopy images showing GFP expression in primary hDFS transfected by nanoinjection using nanoneedles on silicon substrates (top), negative control nanoinjection without GFP mRNA (middle), and lipofection (bottom). (b) Quantification of the fraction of GFP-expressing cells from the fluorescence microscopy experiment shown in (a). *N* = 5 independent biological replicates. One-way ANOVA with post hoc Tukey’s multiple comparisons test; ****p < 0.0001. PLL: poly-l-lysine, Lipo: Lipofectamine Messenger MAX. (c) Flow cytometric quantification of cell fluorescence intensity for cells transfected with nanoneedles on silicon substrates compared to lipofection and the corresponding negative controls. (d) Quantification of the fraction of GFP-expressing cells from flow cytometry. *N* = 3 independent biological replicates. One-way ANOVA with post hoc Tukey’s multiple comparisons test; ****p < 0.0001. (e) Fluorescence microscopy images showing GFP expression in cells transfected with nanoneedles integrated within flexible elastomeric substrates in comparison to their negative control (no mRNA). (f) Quantification of the fraction of GFP-expressing cells from the fluorescence microscopy experiment shown in (e). *N* = 5 independent biological replicates. Mann–Whitney test; **p < 0.01.

We evaluated the efficiency of pSi nanoneedles on PDMS to deliver nucleic acids into primary hDFs in comparison to nanoneedles on silicon substrates. Following the nanoinjection of green fluorescent protein (GFP) mRNA using nanoneedles on silicon substrates, optical microscopy indicated GFP transfection with >90% efficiency, superior to that of lipofectamine (∼50%) (Figures 6a,b and S16). The data were confirmed by flow cytometry (gating strategy in Figure S17). Showing 90% GFP positive cells in nanoinjected samples and up to 40% efficiency with lipofectamine (Figure 6c,d), this indicated that nanoinjection outperforms commercially available chemical nucleic acid delivery for primary human cells.

We then evaluated GFP mRNA nanoinjection into hDFs using nanoneedles integrated within PDMS. The adhesion and survival of cells on the nanoneedles was comparable to that of planar silicon and porous silicon substrates and planar PDMS layers, indicating the good cytocompatibility of our devices (Figure S18). These nanoneedles exhibited a GFP mRNA nanoinjection efficiency of approximately 80%, outperforming lipofectamine transfection (Figure 6e,f). While slightly reduced from the silicon substrate nanoneedles, the nanoneedles on PDMS also confirmed their suitability for nucleic acid delivery by significantly outperforming commercially available transfection reagents in primary cells for highly efficient mRNA nanoinjection. To evaluate the potential contribution to transfection arising from the support layer, we assessed the transfection efficiency of a planar porous silicon layer on PDMS (Figure S19). No transfection was observed for this condition, indicating that the nanoneedles are the key contributors to the transfection efficiency in the PDMS-integrated device.

### Tissue Nanoinjection

**Figure 7:**
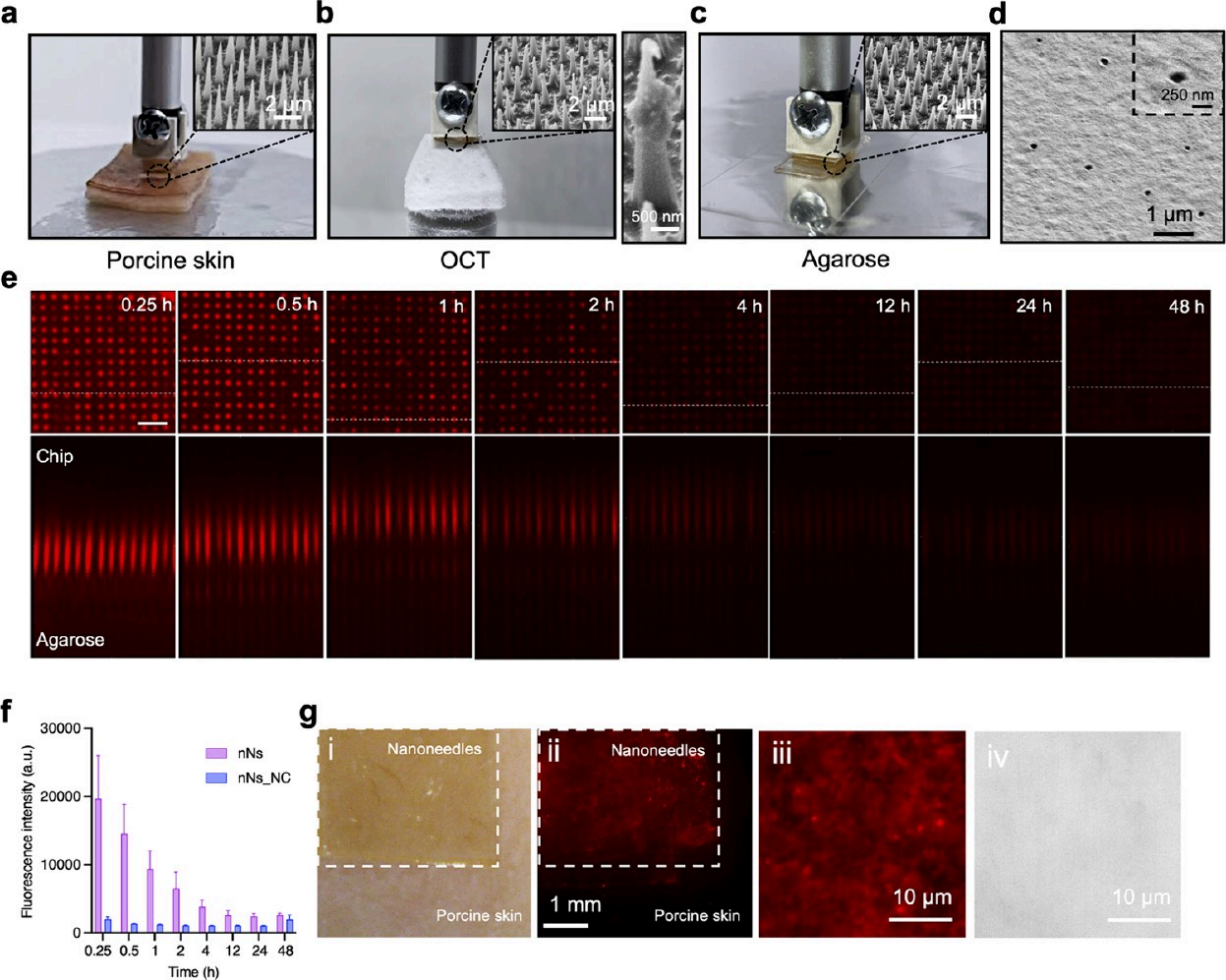
Tissue nanoinjection. (a–d) Optical and SEM images of porous silicon nanoneedles integrated within a flexible elastomer interfacing with (a) porcine skin, showing preserved nanoneedle integrity after interfacing; (b) OCT, showing nanoneedles retaining their integrity (SEM inset) while recovering OCT as an indication of successful interfacing; and (c) agarose thin film, showing nanoneedles retaining their integrity (SEM inset) while producing (d) visible nanopunctures as an indication of successful interfacing. (e) Confocal *XY* and *XZ* orthogonal projections showing the kinetics of payload release from nanoneedles integrated within a flexible elastomer interfaced with a thin agarose film over the course of 48 h. Scale bars: 5 μm. (f) Quantification of the data shown in (e), showing sustained release from nanoneedles over the course of 12 h. (g) (i) bright field and (ii) fluorescence microscopy images showing uniform delivery of nucleic acid across the area of nanoneedle interfacing with porcine skin. High-magnification (iii) fluorescence imaging, showing uniform payload delivery within porcine skin, and (iv) bright field imaging, showing nanoneedles are not retained within the tissues.

We investigated the tissue interfacing of nanoneedles integrated within an elastomeric substrate with a range of surfaces to model their interactions with tissue. SEM analysis of the nanoneedles completed following interfacing with porcine skin (Figure 7a), an optimal cutting temperature (OCT) medium tissue phantom (Figure 7b), and an agarose thin film (Figure 7c) showed that nanoneedles remained on their original substrate and found limited evidence of damage. SEM analysis of the agarose surface (Figure 7d) showed a regular puncture pattern corresponding to the nanoneedle pitch, which was absent in the untreated control (Figure S20).

Nanoinjection of an agarose tissue phantom with nanoneedles carrying a fluorescent payload revealed sustained drug release over the course of 12 h (Figures 7e,f and S21). The nanoinjection of fluorescent nucleic acids in a porcine skin explant (Figure 7g) revealed uniform delivery throughout the tissue in contact with the device (Figure 7g-i–g-iv).

Overall, these data indicated that nanoneedles integrated within flexible devices can effectively interface with tissue for the uniform and sustained topical release of therapeutic payloads.

### Live Animal Nanoinjection

**Figure 8:**
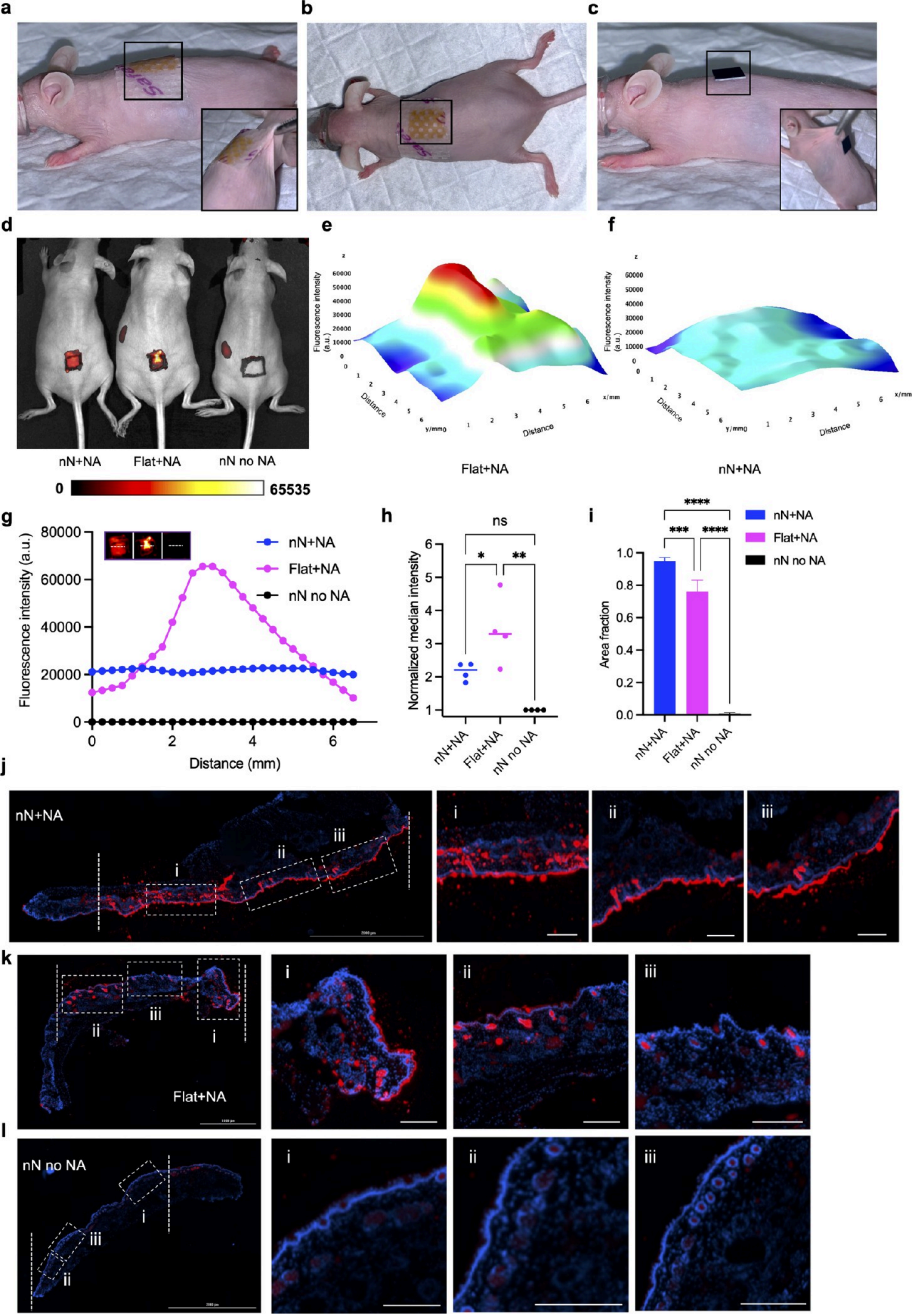
Topical nanoinjection to live mouse skin. (a, b) Images of a Mepilex bandage incorporating nanoneedles applied to the skin of a mouse for nanoinjection. (a) View from the side with inset showing the bandage complying with skin stretching and (b) view from the top. (c) Image of a nanoneedle chip applied to the skin of a mouse for nanoinjection with inset showing the chip failing to comply with skin stretching. (d) Near-infrared live imaging of nude mice nucleic acid (NA) nanoinjection using a fluorescently labeled plasmid loaded on nanoneedles integrated within a flexible elastomer in comparison to a plasmid-loaded flexible elastomer and unloaded nanoneedles. The region of interfacing was labeled using a fluorescent marker to outline the patch shape. (e, f) Maps of the fluorescence intensity distribution for the (e) flat elastomer and (f) nanoneedles, showing higher delivery uniformity from the nanoneedles. (g) Fluorescence intensity signal from a line across the interfacing region for nanoneedles (blue) compared to those for flat elastomer (pink) and empty nanoneedles (black). (h) Normalized median fluorescence intensity originating from the area of nanoinjection for nanoneedles (blue) compared to those for flat elastomer (pink) and empty nanoneedles (black). *N* = 4 independent biological replicates. One-way ANOVA with post hoc Tukey’s multiple comparisons test; *p < 0.05. (i) Quantification of the fraction of the area of interfacing receiving the payload for nanoneedles (blue) compared to those for flat elastomer (pink) and empty nanoneedles (black). *N* = 4 independent biological replicates. One-way ANOVA with post hoc Tukey’s multiple comparisons test; ****p < 0.0001. (j–l) Immunofluorescence microscopy analysis of histological skin sections form the nanoinjection site for (j) nanoneedles, (k) flat elastomer, and (l) empty nanoneedles. The fluorescently labeled nucleic acid is shown in red, and the cell nuclei stained with DAPI are in blue. Nanoneedles display uniform delivery (i–iii) across the area, while large variations in intensity are visible for the flat elastomer. Scale bars: 250 μm.

We applied nanoneedles integrated within a commercial bandage to the skin across the back of a mouse in order to establish their suitability for nanoinjection *in vivo* (Figure 8a,b and Movie S10). The bandage could be applied rapidly and without complications (Movie S10). Once applied, it remained in position and conformed properly to the natural shape of the skin, following the variable curvature across the spinal column (Figure 8a,b and Movie S10). The bandage remained in position and was well tolerated during ambulation, continuing to match the dynamically changing shape of the skin, while the animal did not display signs of discomfort (Movie S11). On the contrary, application of the standard nanoneedle chip could not provide good conformity with the mouse skin and was immediately detached at the onset of motion (Figure 8c and Movies S12 and S13).

We then evaluated the performance of the integrated nanoneedles for the topical nanoinjection of nucleic acids (NA) in live organisms. Live near-infrared (NIR) imaging revealed that nanoneedles uniformly delivered the nucleic acid payload across the curved skin region on the backs of live nude mice (Figure 8d). In contrast, the same device without nanoneedles showed large variations in delivery intensity, and nanoneedles without a payload did not generate a signal. Quantification of the fluorescence intensity across the whole area of interfacing revealed the superior uniformity of nanoneedle delivery with respect to flat bandages (Figure 8e,f), which was confirmed by mapping the fluorescence intensity across a single line through the interfacing region (Figure 8g). The analysis of four independent nanoinjections revealed a narrower intensity distribution for nanoneedles compared with flat bandages (Figure S22). The median delivery intensity was also more narrowly distributed for the nanoneedles (Figure 8h). The fraction of interfacing area that received a payload was higher for nanoneedles at approximately 95%, compared to 77% for flat devices (Figure 8i). Histological analysis of skin sections following nanoinjection further revealed the uniform delivery of nucleic acid across the superficial skin layer interfaced with the nanoneedles (Figure 8j). On the contrary, flat devices exhibited a broader variation in delivery across the area of contact (Figure 8k), and nonloaded nanoneedles did not exhibit fluorescence (Figure 8l). These data indicated that the nanoinjection of nucleic acids with nanoneedle-integrated devices presents high delivery uniformity, high reproducibility, and extensive coverage of the interfaced surface.

**Figure 9:**
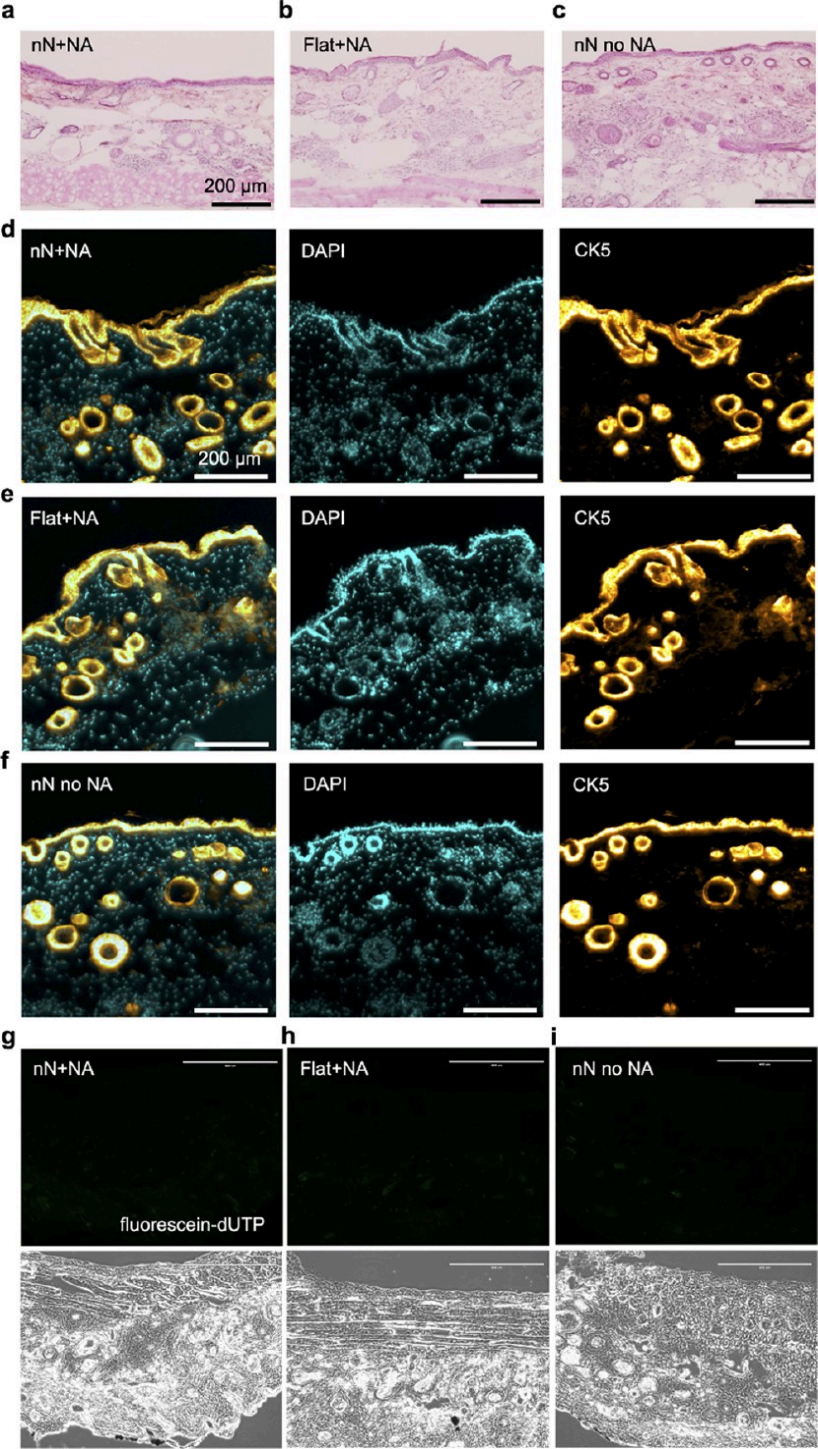
Nanoinjection does not perturb the skin. (a–i) Analysis of histological sections of skin following interfacing with (a, d, g) nanoneedles on flat elastomer loaded with a fluorescent plasmid, (b, e, h) only the elastomer loaded with plasmid, and (c, f, i) nanoneedles without plasmid. (a–c) Hematoxylin and eosin staining to visualize skin structure. (d–f) Immunofluorescence staining with markers for epidermis (CK5, orange hot) and cell nuclei (DAPI, cyan), alongside the fluorescent signal from the nucleic acid payload, to visualize the retained molecular characteristics of skin layers. (g–i) Fluorescent TUNEL staining and corresponding bright field images to assess cell death.

We then evaluated the impact of nanoinjection on the tissue. Hematoxylin and eosin (H&E) histological analysis revealed a preserved skin structure following nanoinjection, comparable to the interfacing of the flat substrate used as the control (Figure 9a–c). Immunohistochemical analysis also revealed a preserved structure and expression of key molecular markers for the epidermis (CK5) following interfacing (Figure 9d–f). Furthermore, terminal deoxynucleotidyl transferase dUTP nick end labeling (TUNEL) staining of the nanoinjected area revealed a cell death pattern analogous to that of the flat control (Figure 9g–i, positive control in Figure S23). These data indicated that nanoinjection, while capable of delivering nucleic acids, did not alter skin structure or induce cell death.

## Conclusions

We have presented a simple and precise transfer approach to integrate well-ordered vertical pSi nanoneedles of arbitrary geometry on a wide range of substrates with different physical and chemical properties, including composition, shape, flexibility, transparency, and bioresorbability. These receiving substrates included key classes of materials employed in biomedical research and real medical devices approved for topical use in the skin, eye, and mucosae. The transfer process was suitable for nanoneedles across a broad range of aspect ratios, porosities, and shapes, demonstrating the necessary versatility to optimize the key parameters to attain nonperturbing intracellular interrogation while preserving their structural stability. This approach requires a support layer to maintain the arrangement of the nanostructures and relies on the generation of a release layer for the peel-off process. The approach also requires a receiving substrate with adhesion capacity to silicon, and substrates lacking such a capacity require additional processing. The integration within transparent devices enabled live high-content imaging to monitor dynamic interactions at the biointerface that are otherwise restricted in opaque substrates. Following transfer, the nanoneedles largely preserved their transfection potential. Nanoneedles integrated within bandages conformed well with the skin of living animals, causing little discomfort. They were effective at tissue nanoinjection, providing uniform delivery and sustained topical release of therapeutics while preserving structural integrity. Nanoinjection of living organisms provided uniform, repeatable, and nonperturbing topical delivery of nucleic acids. Overall, these findings show the ability to manufacture medical devices incorporating nanoneedles that can deliver nucleic acids to tissues and transfect cells with comparable ability but superior versatility to nanoneedles on silicon substrates. This enhanced flexibility can greatly benefit the development of nondisruptive topical nucleic acid therapies, marking a valuable step forward in medical technology.

## Experimental Section

### Fabrication of pSi Nanoneedles

Low-stress epitaxial silicon-rich silicon nitride (120–140 nm) was deposited by low-pressure chemical vapor deposition over 0.01 Ω cm, boron-doped p-type, 100 mm silicon wafers. A 600 nm diameter disk array with a 2 μm pitch was photolithographically patterned on the silicon substrates. Before spin coating the photoresist, the substrate was baked at 200 °C for 20 min in an oven for dehydration. A NR9-250P photoresist was spin-coated onto the silicon wafer, followed by pre-baking at 70 °C for 180 s. Then, it was exposed to UV light under a MA/BA6 mask aligner. A post-bake was then performed at 100 °C for 60 s. After being developed in a 3:1 (v/v) RD_6_/DI (deionized) water solution for 12 s, the patterned substrate was immersed in water to stop development, rinsed with excess water, and then dried under nitrogen jet. Front-end RIE (Oxford Instruments, PlasmaPro NGP80) in CHF_3_ plasma (55 mTorr, 150 W, 50 sccm, 2 min 35 s) was performed to transfer the pattern into the silicon nitride (SiN) layer, followed by washing in acetone and isopropanol and a 10 min oxygen (O_2_) plasma treatment (Diener, 100 W, 0.4 mbar) to strip the photoresist from the substrate.

Following photoresist stripping, the substrate was cleaned in a 1:4 (v/v) mixture of 50% hydrofluoric acid (HF, 20 mL) and DI H_2_O (80 mL) for 2 min, followed by the deposition of Ag from 0.4 M silver nitrate (AgNO_3_) in 50% HF and DI H_2_O (75 mL of DI H_2_O, 20 mL of 50% HF, and 5 mL of 0.4 M AgNO_3_ solution) for 2 min. The substrate was rinsed in DI water and isopropanol and then dried by nitrogen steam. Metal-assisted chemical etching (MACE) was performed with the desired concentration of hydrogen peroxide (H_2_O_2_), e.g., 1% v/v, and HF in DI H_2_O (316 mL of DI H_2_O, 80 mL of 50% HF, and 4 mL of H_2_O_2_) for 7 min 30 s to form porous silicon nanopillars with a diameter of 600 nm and a height of about 7 μm. The composition of the etching solution and MACE duration were tailored to achieve the desired porosities and lengths of nanowires, as described in the Results and Discussion section. The specimen was then washed with DI water and isopropanol and dried under nitrogen steam. Ag stripping was performed by immersing the sample in type TFA etchant for 10 min. The pillars were then shaped into nanoneedles by reactive ion etching in SF_6_ (20 sccm) plasma. Etching parameters were tuned to achieve the desired shape, as described in the Results and Discussion section. The substrate was diced into chips of desired size (DAD3230, DISCO dicing saw), and the nanoneedles were oxidized for 10 min with O_2_ plasma at 100 W RF power and 0.4 mbar immediately prior to use.

### Transfer Process of pSi Nanoneedles

The polymerized PDMS substrate (Sylgard 184, 20:1 v/v base to curing agent ratio, thickness of 1 mm) was cast and cured before the transfer process. The water-soluble tape (3M, Wave Solder Tape) was adhered to the top of the nanostructure array, contacting the free ends (i.e., tips). The nanostructure array, together with the porous layer, was separated from the donor substrate by peeling, that is, by pulling the tape to apply a force at the interface between the porous layer and the detachment layer. The water-soluble tape was placed in contact with the surface of the receiving substrate, and the assembly was immersed in DI water at 60 °C for 10 min, resulting in full tape dissolution and integration of the nanostructures within the receiving substrate. Transferring pSi nanoneedles onto non-intrinsically adhesive substrates such as polypropylene tubes, sharp, convex structures, and poly(lactic acid) involved first depositing a PDMS thin film over their surface as an adhesion layer.

### *Ex Vivo* Nanoinjection

Porous Si nanoneedle arrays on PDMS were first treated with oxygen plasma (100 W) for 10 min (ZEPTO-W6, Diener electronic) incubated with 0.1 mg/mL poly-l-lysine (25988-63-0, Sigma-Aldrich) for 30 min and rinsed three times in ddH_2_O. The nanoneedles were incubated with 10 μM fluorophore-conjugated nucleic acid dT30-TEX615 (Integrated DNA Technologies, Inc.) in TE buffer for 1 h at room temperature in the dark. Substrates were washed in TE buffer and air-dried. The freshly prepared nanoneedles were compressed onto porcine skin for 10 s as described above and were left in place for 1 h before removal. The skin was hard-mounted (ProLong Gold Antifade Mountant, Invitrogen) onto coverslips. A tile-scan image across the area of interfacing was generated using a Leica DMi8 microscope with a 20× 0.4 NA air objective, and high-magnification images were captured with a 63× 1.2 NA water objective.

### *In Vivo* Nanoinjection

All animal protocols and experiments were undertaken in accordance with the University of Minnesota Institutional Animal Care and Use Committee (IACUC) guidelines (protocol number: 2106-39156A). Female, hairless mice (*n* = 4, 8 weeks old, SKH1-Hrhr, Charles River Laboratories, U.S.) were used for *in vivo* testing. Mice were housed using standard, small-animal research conditions with a 12 h light/dark cycle, free access to food and water, and temperatures ranging from 22 to 24 °C. Mice were anesthetized with inhaled isoflurane anesthesia and 2.5–4% isoflurane delivered in O_2_ (1 L/min) within a 1 L induction chamber, followed by a nose cone to maintain sedation. A Tegaderm adhesive bandage (3M, U.S.) was applied to the lower dorsal area of the mice and pulled off to slightly abrade the skin. The substrates were applied to the lower dorsal area of the mice using tweezers. Chips were pressed firmly onto the skin for 10 s and remained on-skin for 2 min. Chips were removed, and the mice were imaged with the IVIS Spectrum *in vivo* imaging system (PerkinElmer, U.S.).

## Supporting Information Available

Additional experimental results, details, materials, and methods (PDF)
Movie S1: successful transfer of the support layer and nanopillar array to water-soluble tape (MP4)
Movie S2: unsuccessful transfer of the nanopillar array to water-soluble tape due to the absence of a release layer (MP4)
Movie S3: 90° peel test of detaching the nanoneedles from the Si wafer (with release layer) (MOV)
Movie S4: uniaxial stretching of rectangular PDMS substrate with nanoneedles, showing their flexibility and stretchability (MP4)
Movie S5: uniaxial stretching of square PDMS substrate with nanoneedles, showing their flexibility and stretchability (MP4)
Movie S6: live high-content imaging of hDFs cultured on nanoneedles over PDMS in bright field (MP4)
Movie S7: live high-content imaging of hDFs cultured on nanoneedles over PDMS in fluorescence (MP4)
Movie S8: live high-content imaging of hDFs cultured on a flat PDMS surface (control) in bright field (MP4)
Movie S9: live high-content imaging of hDFs cultured on a flat PDMS surface (control) in fluorescence (MP4)
Movie S10: application of a commercial bandage (with nanoneedles integrated within) to the skin of the back of a mouse (MOV)
Movie S11: mouse walking around with bandage, which was well tolerated and remained in position (MOV)
Movie S12: application of standard nanoneedle chip to the skin of the back of a mouse (MOV)
Movie S13: mouse walking around with standard nanoneedle chip, which immediately detached at the onset of motion (MOV)

## Supplementary Material

nn4c00206_si_003.mp4

nn4c00206_si_004.mov

nn4c00206_si_010.mp4

nn4c00206_si_011.mov

nn4c00206_si_012.mov

nn4c00206_si_013.mov

nn4c00206_si_014.mov
