# Supplementary material for: Integrating Porous Silicon Nanoneedles within Medical Devices for Nucleic Acid Nanoinjection
Source: ACS Nano. 2024 May 10;18(23):14938–53. doi: 10.1021/acsnano.4c00206 (PMC11171749; doi:10.1021/acsnano.4c00206)
Supplement: Supplementary file 1 — nn4c00206_si_001.pdf [file nn4c00206_si_001.pdf]

Supporting Information

Integrating Porous Silicon Nanoneedles within Medical Devices for Nucleic Acid Nanoinjection

Cong Wang[1,2], Chenlei Gu[1,2], Courtney Popp[3], Priya Vashisth[1], Salman Ahmad Mustfa[1], Davide Alessandro Martella[1,2], Chantelle Spiteri[1], Samuel McLennan[1], Ningjia Sun[1], Megan Riddle[3], Cindy R. Eide[3], Maddy Parsons[4], Jakub Tolar[3,5], John A. McGrath[6], Ciro Chiappini[1,2*]

1. Centre for Craniofacial and Regenerative Biology, King's College London, SE1 9RT, London, UK.
2. London Centre for Nanotechnology, King's College London, WC2R 2LS, London, UK.
3. Department of Pediatrics, Medical School, University of Minnesota, Minneapolis, MN 55455, USA
4. Randall Centre for Cell and Molecular Biophysics, King's College London, London, UK
5. Stem Cell Institute, University of Minnesota, Minneapolis, MN 55455, USA
6. St John's Institute of Dermatology, King's College London, UK

*Correspondence to: ciro.chiappini@kcl.ac.uk

Table of Contents

METHODS/EXPERIMENTAL 3

Fabrication of pSi nanoneedles 3
Generation of support and release layer: 4
Transfer process of pSi nanoneedles: 4
Mechanical testing: 4
Tuning nanoneedle transparency: 5
Nanoneedle transparency: 5
Nanoneedle transmittance: 5
UV-Vis absorption spectroscopy: 5
Nanoneedle integrity: 5
Ex vivo interfacing: 5
Ex vivo nanoinjection: 6
Release kinetics: 6
hDF cell culture: 6
Biointerface visualization: 7
High-content live imaging: 7
mRNA nanoinjection of hDF: 7
GFP expression microscopy analysis: 7
GFP flow cytometry analysis: 7
Cell attachnent and cytotoxicity assay of hDF: 8
Plasmid loading: 8
In vivo nanoinjection: 8
IVIS imaging: 9
Skin Immunohistochemistry: 9
TUNEL Staining: 10
Analysis of in vivo delivery: 10
Statistical analysis: 10
Scanning electron microscopy (SEM) characterization: 11

SUPPLEMENTARY REFERENCES 11

SUPPLEMENTARY FIGURES 12

Methods/Experimental

Fabrication of pSi nanoneedles: Low-stress epitaxial silicon rich silicon nitride (120-140 nm) was deposited by low-pressure chemical vapour deposition over 0.01 Ω-cm, boron-doped p-type, 100 mm silicon wafers. A 600-nm-diameter disk array with 2 µm pitch was photo-lithographically patterned on silicon substrates. Before spin coating photoresist, the substrate was baked at 200 °C for 20 min in oven for dehydration. NR9-250P photoresist was spin-coated onto the silicon wafer, followed by pre-baking at 70 °C for 180 s, before being exposed to UV light under a mask aligner of MA/BA6. Post-bake was then performed at 100 °C for 60 s. After being developed in a 3:1 (v/v) RD_6: DI (de-ionized) water solution for 12 s, the patterned substrate was immersed in water to stop development and rinsed with excess water, then dried under nitrogen jet. The front-end RIE (Oxford Instruments, PlasmaPro NGP80) in CHF_3 plasma (55 mTorr, 150 W, 50 sccm, 2 min 35 s) was performed to transfer the pattern into the silicon nitride (SiN) layer, followed by washing in acetone and isopropanol and 10 min oxygen (O_2) plasma treatment (diener, 100 w, 0.4 mbar) to strip the photoresist from the substrate.

Following photoresist stripping, the substrate was cleaned in a 1:4 (v/v) mixture of 50% hydrofluoric acid (HF, 20 ml) and DI H_2O (80 ml) for 2 min, followed by deposition of Ag from 0.4 M silver nitrate ($AgNO_3$) in 50% HF and DI H_2O (75 ml DI H_2O, 20 ml 50% HF and 5 ml 0.4 M $AgNO_3$ solution) for 2 min. The substrate was rinsed in DI water and isopropanol, then dried by nitrogen steam. Metal-assisted chemical etch (MACE) was performed with the desired concentration of hydrogen peroxide (H_2O_2), *e.g.*, 1 % v/v, and HF in DI H_2O (316 ml DI H_2O, 80 ml 50% HF and 4 ml H_2O_2) for 7 min 30 s to form porous silicon nanopillars with 600 nm diameter and about 7 µm height. The composition of the etching solution and MACE duration were tailored to achieve desired porosities and length of nanowires, as described in the results. The specimen was then washed with DI water and isopropanol, dried under nitrogen steam. Ag stripping was performed by immersing in Type TFA etchant for 10 min. The pillars were then shaped into nanoneedles by reactive ion etching in in SF_6 (20 sccm) plasma. Etching parameters were tuned to achieve desired shape, as described in the results. The substrate was diced into chips of desired size (DAD3230, DISCO Dicing Saw), and the nanoneedles were oxidized by 10 min O_2 plasma at 100W RF, 0.4 mBar immediately prior to use.

Generation of support and release layer: Following formation of the pSi nanostructures by MACE, electrochemical etch (EC) was used to generate the support and release layers[1]. An aluminium (Al) electrode film was contacted to the backside of the Si wafer. The frontside of the wafer was exposed to 1:3 (v/v) mixed electrolyte solution of 50% HF (65 ml) and 100% ethanol (195 ml) in a Teflon anodization cell. A DC power supply (KEYSIGHT, E36231A) was connected with the anode to the aluminium film and the cathode to the platinum (Pt) electrode mesh immersed in the electrolyte solution[2] (Figure S24). A current density of 34 mA cm^{-2} was applied for 60 s to generate a 600 nm thick of support layer underlying the Si nanostructure arrays. The release layer was formed underneath the support layer by applying the current density of 101 mA cm^{-2} for 2 s. The substrate was then removed from the anodization cell, rinsed with DI water and ethanol, dried by nitrogen steam.

Transfer process of pSi nanoneedles: The polymerized PDMS substrate (Sylgard 184, 20:1 v/v base and curing agent ratio, thickness 1 mm) was cast and cured before the transfer process. The water-soluble tape (3M Adhesive Tapes, WAVE SOLDER TAPE) was adhered to the top of the nanostructure array, contacting to the free ends (*i.e.*, tips). The nanostructures array together with the porous layer were separated from the donor substrate by peeling, that is, by pulling the tape to apply a force at the interface between the porous layer and the detachment layer. The water-soluble tape was placed in contact with the surface of the receiving substrate and the assembly was immersed in DI water at 60 °C for 10 min resulting in full tape dissolution and integration of the nanostructures within the receiving substrate. Transferring pSi nanoneedles onto non-intrinsically adhesive substrates such as polypropylene tube, sharp, convex structures, and poly-lactic acid, involved first depositing a PDMS thin film over their surface as adhesion layer.

Mechanical testing: The nanoneedles carried on the water soluble tape, were placed in contact with a glass coverslip spin-coated with a PDMS thin film (SPIN150i, APT GmbH) at 2250 rpm for 140 s with 50 rpm/s spin acceleration. The assembly was compressed using a custom force-feedback linear actuator for 10 s. After compression, the tape was dissolved according to the transfer process method, and the integrity of the nanoneedle array was assessed by using scanning electron microscopy imaging (Carl Zeiss XB1540 Crossbeam SEM/FIB).

Tuning nanoneedle transparency: Nanostructures were prefabricated on Si substrate and transferred onto PDMS with a 600 nm support layer. The device underwent reactive ion etching in SF_6 (20 sccm) plasma at 200 W, 100 mTorr, 100 mTorr strike pressure for durations between 150 s and 280 s to achieve layer thicknesses between 200 nm and 0 nm.

Nanoneedle transparency: The chips were first imaged using a USB digital microscope (196-4074, RS PRO). The mean grayscale values of three randomly selected ROIs (50x50 pixels) from the nanoneedles and PDMS region were calculated. Transparency level was determined by normalizing the mean grayscale values of the nanoneedles region to the PDMS region.

Nanoneedle transmittance: Samples were hard-mounted onto coverslips overnight (ab104139, Abcam). Three bright field images of each sample were acquired by a Leica DMi8 microscope with a 63X 1.2 NA water objective. For transmittance, a custom-MATLAB (R2021a) script was generated to separately measure the opacity of the substrate and nanoneedles. Five regions (200x200 pixels) were randomly selected from each image for analysis. The digital image processing script performed local thresholding, particle size exclusion, and segmentation to extract the grayscale value of every single nanoneedle and the entire substrate. The transmittance of nanoneedles and substrate was subsequently calculated by normalizing their mean grayscale values to the value of the PDMS substrate in a region without nanoneedles.

UV-Vis absorption spectroscopy: Flexible nanoneedles with different thicknesses of support layer (T_s) integrated within PDMS and a PDMS layer were first cut to fit a UV-transparent microplate (Greiner UV-Star® microplate, 675801). All chips were treated with oxygen plasma (ZEPTO-W6, Diener electronic) at 100W, 0.4 mbar for 10 min before transferring into the microplate and 100 µL DI water was added to each well. The absorbance spectrum (220 nm to 1000 nm) was measured using a CLARIOstar Plus plate reader (using CLARIOstar v5.21 and MARS v3.20 software).

Nanoneedle integrity: The integrity of nanoneedles was evaluated manually by counting nanoneedles in Fiji on the segmented images acquired for nanoneedle transmittance.

Ex vivo interfacing: The pure optimal cutting temperature (OCT) compound block was prepared using flash freezing in plastic molds. Porcine skin was sourced locally, shaved and rinsed in deionized water. The agarose mattress (1.4% in ddH_2O) was prepared freshly by

casting between two coverslips at 500 µm thickness. Porous Si nanoneedle arrays on PDMS were interfaced with a controlled force, using a custom force-feedback compression device.

Ex vivo nanoinjection: Porous Si nanoneedle arrays on PDMS were first treated by oxygen plasma (100W) for 10 min (ZEPTO-W6, diener electronic) incubated with 0.1 mg/mL Poly-L-Lysine (25988-63-0, Sigma Aldrich) for 30 min and rinsed three times in ddH_2O. The nanoneedles were incubated with 10 µM the fluorophore-conjugated nucleic acid dT30-TEX615 (Integrated DNA Technologies, Inc), in TE buffer for 1 h at room temperature in the dark. Substrates were washed in TE buffer and air-dried. The freshly-prepared nanoneedles were compressed onto the porcine skin for 10 s as described above and were left in place for 1 h before removal. The skin was hard-mounted (ProLong™ Gold Antifade Mountant, Invitrogen) onto coverslips. A tile-scan image across the area of interfacing was generated using a Leica DMi8 microscope with a 20X 0.4 NA air objective, and high-magnification images were captured with a 63X 1.2 NA water objective.

Release kinetics: Porous Si nanoneedle arrays on PDMS were treated by oxygen plasma (100W) for 10 min (ZEPTO-W6, diener electronic), then incubated with 0.05 mg/mL rhodamine B (A13572, Alfa Aesar) for 1 h at room temperature in the dark. The samples were rinsed and air dried. The nanoneedles were compressed onto the agarose for 10 s as described above, left in place and soft-mounted using KPL mounting medium (5570-0005, SeraCare). The samples were imaged by Z-stack confocal microscopy (Zeiss LSM 980) with a 20X 0.8 NA air objective. The maximum projected Z stack images were analysed using the custom MATLAB script, described in the nanoneedle transmittance and integrity method. The script evaluated the fluorescence intensity from nanoneedles over time. Orthogonal projections were generated for visualization.

hDF cell culture: Devices were sterilized with 70% v/v ethanol in deionized water for 1 h, dried and UV irradiated for 20 min. They were placed at the bottom of a 24-well plate and rinsed three times with PBS. Primary human dermal fibroblast cells were seeded over the devices at a density of $1X10^5$ cells/well. Cells were cultured in high glucose DMEM – GlutaMAX™ (31966-021, Gibco) supplemented with 10% fetal bovine serum and 1% penicillin/streptomycin and grown in a humidified incubator at 37 °C with 5% CO_2.

Biointerface visualization: At the desired time points (2 h, 4 h and 72 h), cells were fixed using 4% paraformaldehyde (PFA) for 10 min. For confocal microscopy analysis, the devices were hard mounted on coverslips and imaged using a ZEISS LSM 980. For scanning electron microscopy (Carl Zeiss XB1540 Crossbeam SEM/FIB), samples were fixed with 4% paraformaldehyde for 10-15 min at room temperature and dehydrated in ethanol gradients 30%, 50%, 70%, 90%, 95% and 100%.

High-content live imaging: Cells were transfected from nanoneedles using 1 ug GFP mRNA (TriLink) 12 hours prior to live imaging. Images were acquired on a Leica DMI8 inverted microscope with a 20X 0.4 NA lens. During live imaging, cells were incubated with 5% carbon dioxide at 37 °C and imaged at 1-hour intervals over 36 hours with bright field and FITC channel (excitation/emission 475/527 nm) with 200 ms exposure.

Cell area and aspect ratio were measured using a custom Fiji script that performed batch process thresholding, masking, and analysis with defined parameters and exclusion principles (Figure S25). Cell speed was measured using Cell Profiler's centroid tracking pipeline (Figure S26), which performed batch thresholding and masking and centroid tracking of unique cells over time series.

mRNA nanoinjection of hDF: All devices were treated with O_2 plasma (ZEPTO-W6, diener electronic) 100W, 0.4 mbar for 10 min. Sterilized devices were placed in 24 well plate and incubated with 200 ul of 0.1 mg/ml poly-L-lysine (PLL) for 1 hour in sterile conditions. Needles were washed with PBS (3 times). 1 ug eGFP mRNA (TriLink) was added onto the devices for 30 min in 100 ul sterile PBS. Human dermal fibroblasts then were seeded on nanoneedles at 100,000 cells/well for transfection. For lipofectamine messenger max (Thermo Fisher) samples, the manufacturer's transfection guidelines were followed.

GFP expression microscopy analysis: The samples were fixed using 4% PFA for 10 min. After fixing, samples were washed with PBS (3 times). Cells were stained with WGA-647 (Thermo Fisher) with 1:1000 dilution. Samples were mounted on coverslip using fluoroshield media containing DAPI (Abcam). Imaging was acquired on Leica DMI8 inverted microscope using appropriate filters and quantified manually.

GFP flow cytometry analysis: Transfected hDF cells were washed with phosphate buffer saline (PBS). Cells were dissociated using 0.25% trypsin and re-suspended in 1 ml stain buffer

(BD Biosciences). LSR Fortessa using FACS diva software (BD Biosciences) were used to quantify fluorescence intensity in the cells and a minimum of 10,000 events per condition were analysed. Event selection and fluorescence quantification were performed using FlowJo according to the gating strategy shown in Figure S17.

Cell attachment and cytotoxicity assay of hDF: All devices were treated with oxygen plasma (ZEPTO-W6, diener electronic) 100W, 0.4 mbar for 10 min. Sterilized devices were placed in 24 well plate and incubated with 200 ul of 0.1 mg/ml poly-L-lysine (PLL) for 1 hour in sterile conditions. The devices were washed with PBS (3 times). hDF cells were seeded on the devices at 100,000 cells/well. The devices were moved to a new well position after a 24-hour culture time and the cells were dissociated using 0.25% trypsin and re-suspended in 1 ml stain buffer (BD Biosciences). An aliquot was taken from each sample and counted using a haemocytometer. The resulting cell attachment was calculated relative to the initial seeding density and accounting for the size of the devices. Cell cytotoxicity was performed by staining cells with Propidium Iodide (manufacturer) with 1:3000 dilution. LSR Fortessa using FACS diva software (BD Biosciences) was used to quantify fluorescence intensity in the cells and a minimum of 10,000 events per condition were analysed. Event selection and fluorescence quantification were performed using FlowJo.

Plasmid loading: Nanoneedles on PDMS and flat PDMS substrates were oxidized with 300 W, 300 mT pressure, and 99 sccm O_2 flow for 10 min. Samples were then treated with Poly-L-Lysine (0.1 mg/ml, 100 µl, Sciencell Research Laboratories, USA) for 1 hour at room temperature and washed three times with 1x PBS (ThermoFisher Scientific, USA). A plasmid (NM_000094, Origene) was tagged with CY5 using the Mirus Label-it kit according to manufacturer's instructions. Following washing step, the CY5-tagge plasmid was added to the substrates (60 µl, 1µg/60 µl) and incubated at room temperature, covered from light, for 30 min. Nanoneedle on PDMS substrates processed in the same fashion but without CY5-tagged plasmid were also generated as controls. The substrates without plasmid were incubated with water. Following incubation, chips were applied to mice.

In vivo nanoinjection: All animal protocols and experiments were undertaken in accordance with the University of Minnesota Institutional Animal Care and Use Committee (IACUC) guidelines (protocol number: 2106-39156A). Female, hairless mice (n = 4, 8 weeks old, SKH1-Hrhr, Charles River Laboratories, USA) were used for in vivo testing. Mice were housed using standard, small-animal research conditions with a 12-hour light: dark cycle, free access to food and water, and temperatures ranging from 22-24° C. Mice were anesthetized with

inhaled isoflurane anaesthesia and 2.5-4% isoflurane delivered in O_2 (1 litres/min) within a 1-liter induction chamber followed by a nose cone to maintain sedation. A Tegaderm adhesive bandage (3M, USA) was applied to the lower dorsal area of mice and pulled off to slightly abrade the skin. The substrates were applied to the lower dorsal area of the mice using tweezers. Chips were pressed firmly onto the skin for 10 s and remained on-skin for 2 min. Chips were removed and mice were imaged with the IVIS Spectrum In Vivo Imaging System (PerkinElmer, USA).

IVIS imaging: Fluorescence of the CY5-tagged plasmid was measured with the IVIS Spectrum In Vivo Imaging System (PerkinElmer, USA) after removing the nanoneedle chip. The fluorescence was captured with an exposure time of 1 s and was filtered using a red fluorophore cyanine 5 (CY5) excitation filter of 635 to 655 nm and emission filter of 658 to 678 nm. Following imaging, data were analysed by Living Image software (PerkinElmer, USA).

Skin Immunohistochemistry: Skin from mice was frozen in optimal cutting temperature (OCT, Sakura Finetek USA, Torrance,CA). The blocks were sectioned at 6 microns on a cryostat. Slides were fixed in 4% PFA for 5 min at room temperature. Slides were washed twice with 1x PBS (ThermoFisher Scientific, USA) and coverslipped with Vibrance DAPI, 4,6-diamidino-2-phenylindole, (Vector Labs, Burlingame, CA). Slides were imaged on the BioTek Cytation 5 Cell Imaging Multimode Reader (Agilent, USA).

To examine the integrity of the skin following nanoneedle chip application, H&E was performed in addition to IF staining of normal skin markers. Slides were fixed in 3.7% PFA/10% Sucrose solution for 5 min at room temperature. Slides were washed 2 times with 1x PBS (ThermoFisher Scientific, USA) then permeabilized with 0.2% Triton X for 5 min. Slides were blocked with 3% BSA for 1 hour. Primary antibodies Cytokeratin 5 (BioLegend, San Diego, CA 1:500) and Vimentin (Abcam, Waltham, MA 1:200) were applied for 1 hour at room temperature. Slides were washed with 1x PBS (ThermoFisher Scientific, USA). Secondary donkey anti-rabbit Cy3 (Jackson Immunoresearch, West Grove, PA 1:500) and goat anti-chicken IgY Alexa 488 (Invitrogen, Waltham, MA 1:800) were applied for 1 hour at room temperature. Slides were washed with 1x PBS (ThermoFisher Scientific, USA) and coverslips applied with Vibrance DAPI, 4,6-diamidino-2-phenylindole, (Vector Labs, Burlingame, CA). Slides were imaged on the BioTek Cytation 5 Cell Imaging Multimode Reader (Agilent, USA).

TUNEL Staining: Slides were fixed with 4% PFA for 20 min, washed for 30 min in 1x TBS, then permeabilized with 0.1% Triton X for 2 min. Slides were washed again with 1x TBS and then TUNEL-Mix (In Situ Cell Death Detection Kit, Fluorescein, Sigma Aldrich, St. Louis, MO) was added to slides and incubated for 1 hour in the dark at room temperature. Slides were again washed with 1x TBS and Propidium iodide 1ug/ml was applied for 5 min. Slides were washed with 1x TBS and coverslipped with Immu-Mount (Thermo Scientific, Waltham, MA). Slides were imaged on the EVOS Cell Imaging System (ThermoFisher Scientific, USA). To induce DNA strand breaks for a positive control, murine skin tissue slides (8 weeks old, SKH1-Hrhr, Charles River Laboratories, USA) were fixed with 4% PFA for 20 min and permeabilized with 0.1% Triton X for 2 min followed by incubation with Dnase I Recombinant Grade 1 (3,000 U/ml Roche, USA) in 50 mM Tris-HCl, pH 7.5, 10 mM MgCl2, and 1mg/ml BSA for 20 min at RT prior to Tunel labelling procedure as reported above.

Analysis of in vivo delivery: The Near-infrared imaging (NIR) images were analysed using a custom Fiji script to extract the fluorescence intensity across the entire area of interfacing (interactive 3D surface plot) and a selected line through the interfacing region. The script also returned the median delivery intensity and the area fraction for each sample analysed. A custom MATLAB (R2021a) script was used to outline the area of interfacing, extract the fluorescent intensity for each peak, normalise the data and plot the intensity distribution. Normalisation was performed by first aligning the median of all individual experiments. This allowed comparing the distribution of intensities across samples discounting for the variation of their median intensities, which was larger for flat samples. The minimum value of the entire dataset was then set to 0 for ease of visualisation.

Statistical analysis: Graphs throughout the manuscript display independent measurements as individual datapoints. Unless otherwise specified in the caption, for each group within the analysis the height of the bar is the average of the independent measurements and the error bar their standard deviation. Statistical analysis was only performed when at least three independent samples had been measured. The number of independent measurements, the statistical methods employed, and the significance values obtained are reported in the relative figure caption for each graph.

Scanning electron microscopy (SEM) characterization: All SEM images were captured using Carl Zeiss XB1540 Crossbeam SEM/FIB at tilted 45°. An Edwards sputter (Emscope SC 500) was used to coat Au for required samples.

Supplementary References

1. Sailor, M. J. Porous Silicon in Practice. In *Preparation, Characterization and Applications*; Wiley, Verlag GmbH & Co. KgaA, Weinheim, 2012; pp 1–42.
2. Tasciotti, E.; Martinez, J.; Chiappini, C.; Bhavane, R.; Ferrari, M. Methods in Bioengineering. In *Nanoscale Bioengineering and Nanomedicine*; Rege, K., Medintz, I. L., Eds.; Artech House, 2009; pp 237–291.

Supplementary Figures

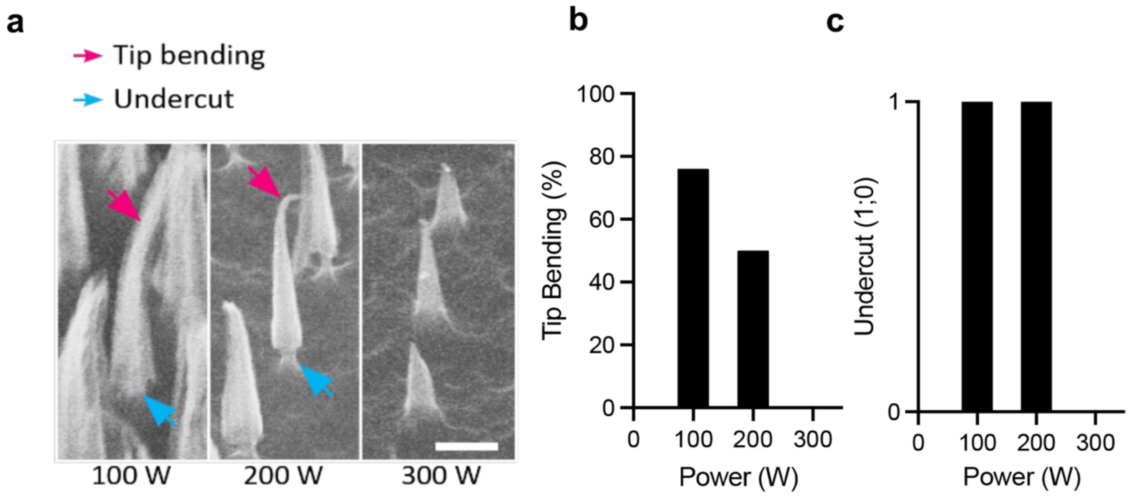

Figure S1. Reactive Ion Etching control of nanoneedle shape. (a) SEM images showing the structural instabilities: tip bending (magenta arrows) and undercut (cyan arrows). Scale bars: 1 µm. (b-c) Quantification of the structural instabilities: percentage of tip bending and presence of undercut as a function of RIE RF power. The power increase was associated with a decrease in percentage of bent tips, and no undercut was present for 300 W. Power of 200 W and 300 W showed higher anisotropy of the process and thus higher structural stability of nanoneedles.

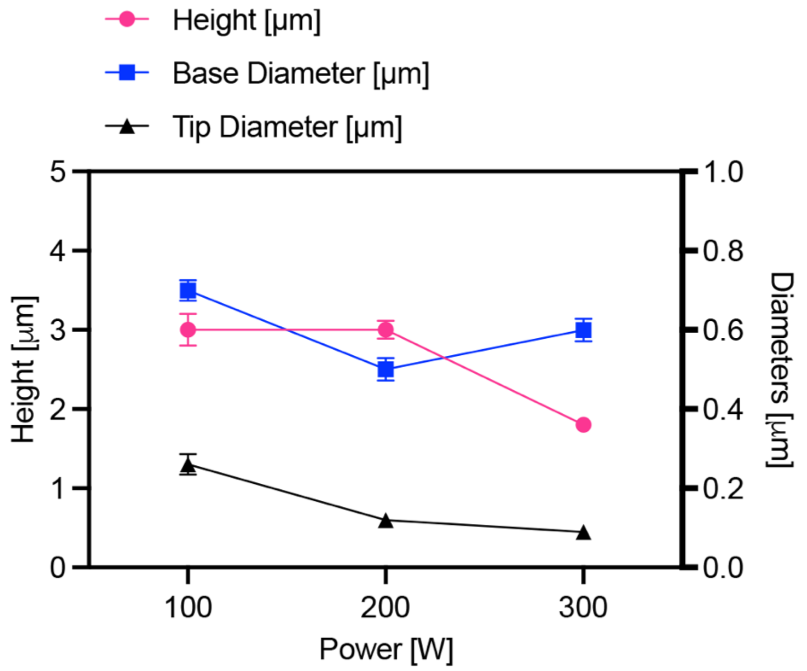

Figure S2. Plot of the nanoneedles height, base diameter and tip diameter calculated from SEM images, with error bars showing the standard error of the mean.

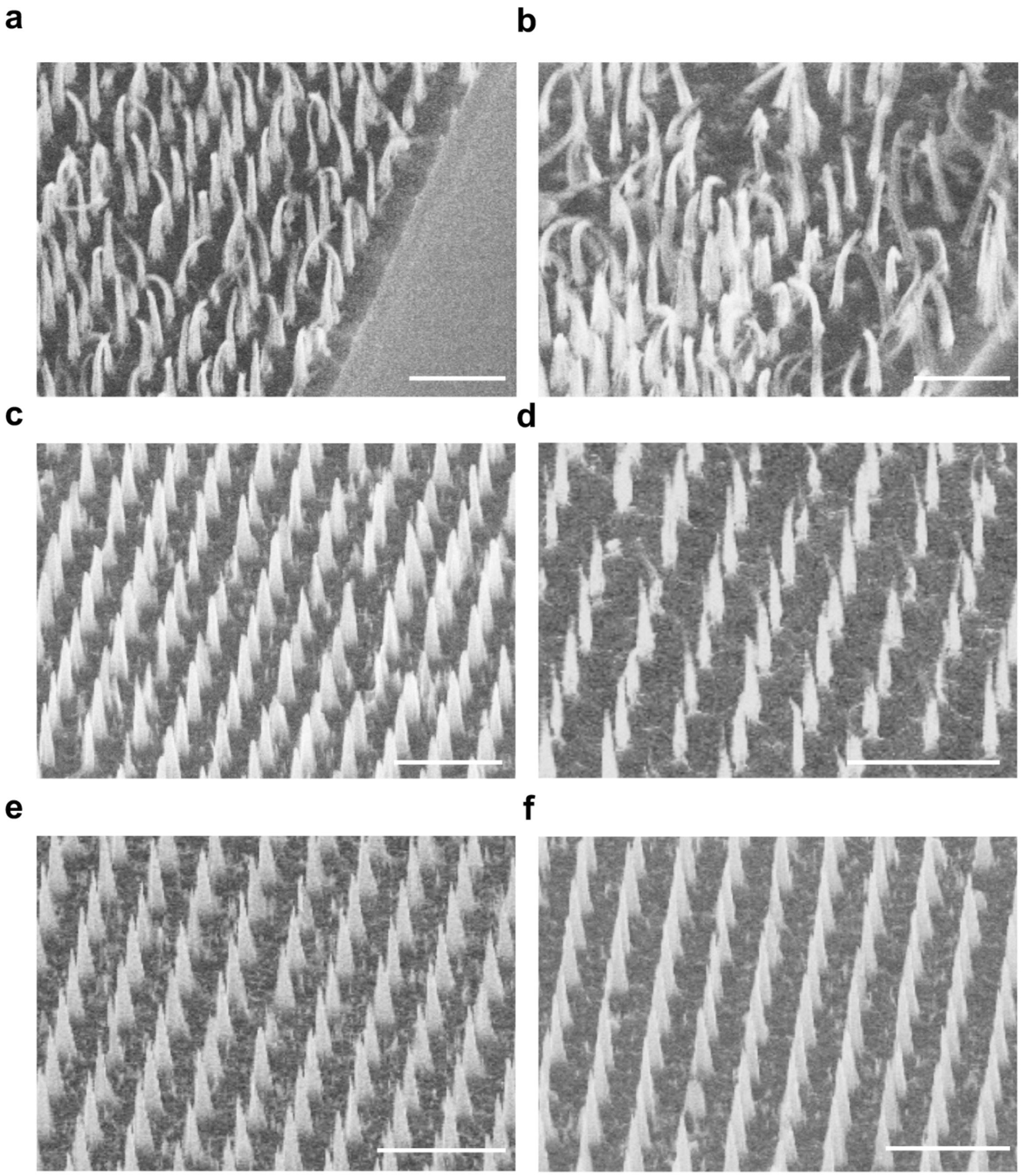

Figure S3. SEM images of nanoneedles after RIE at (a-b) 100 W, 165 s, with different gas pressure of (a) 50 mTorr and (b) 100 mTorr. (c-d) 200 W, 150 s, with gas pressure of (c) 50 mTorr and (d) 100 mTorr. (e-f) 300 W, 120 s at gas pressure of (e) 50 mTorr and (f) 100 mTorr. Scale bars: 5 µm.

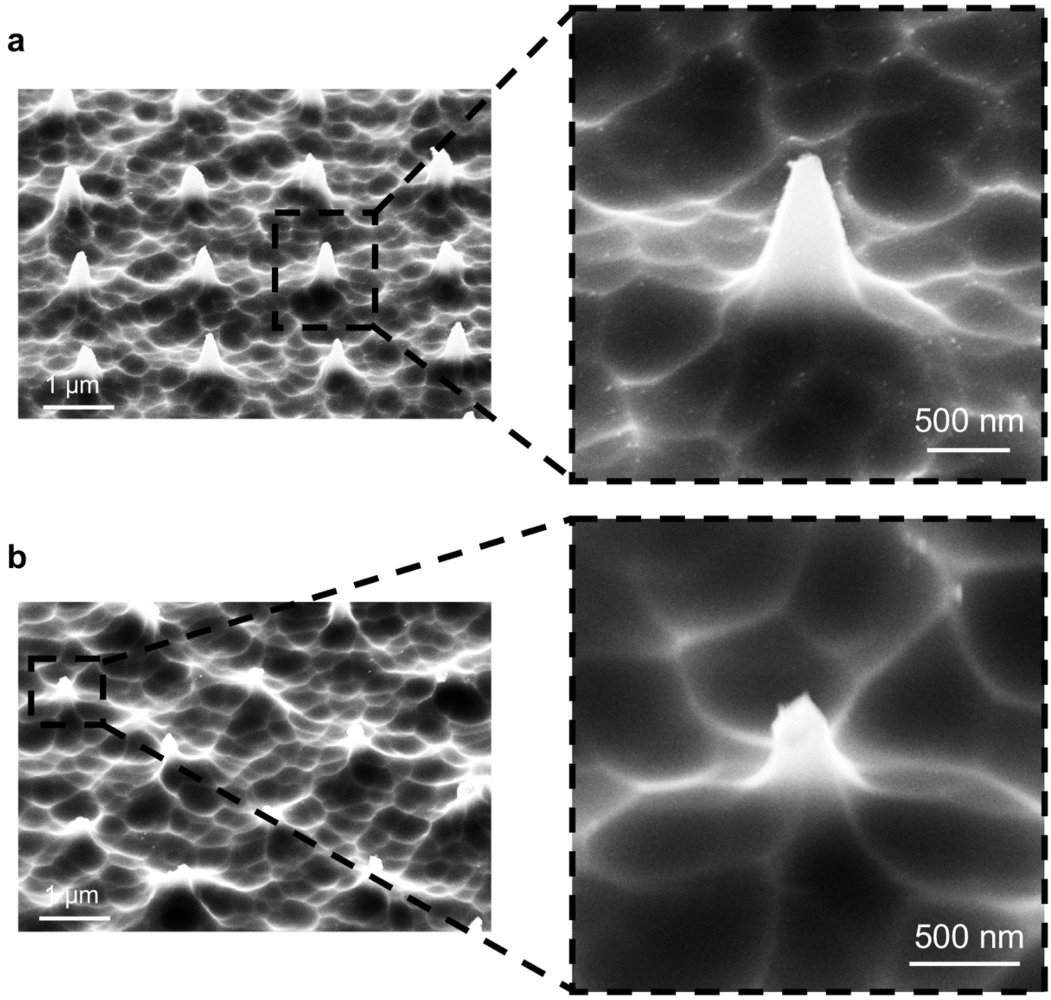

Figure S4. SEM images showing pSi nanoneedles with (a) 1 µm and (b) 500 nm height.

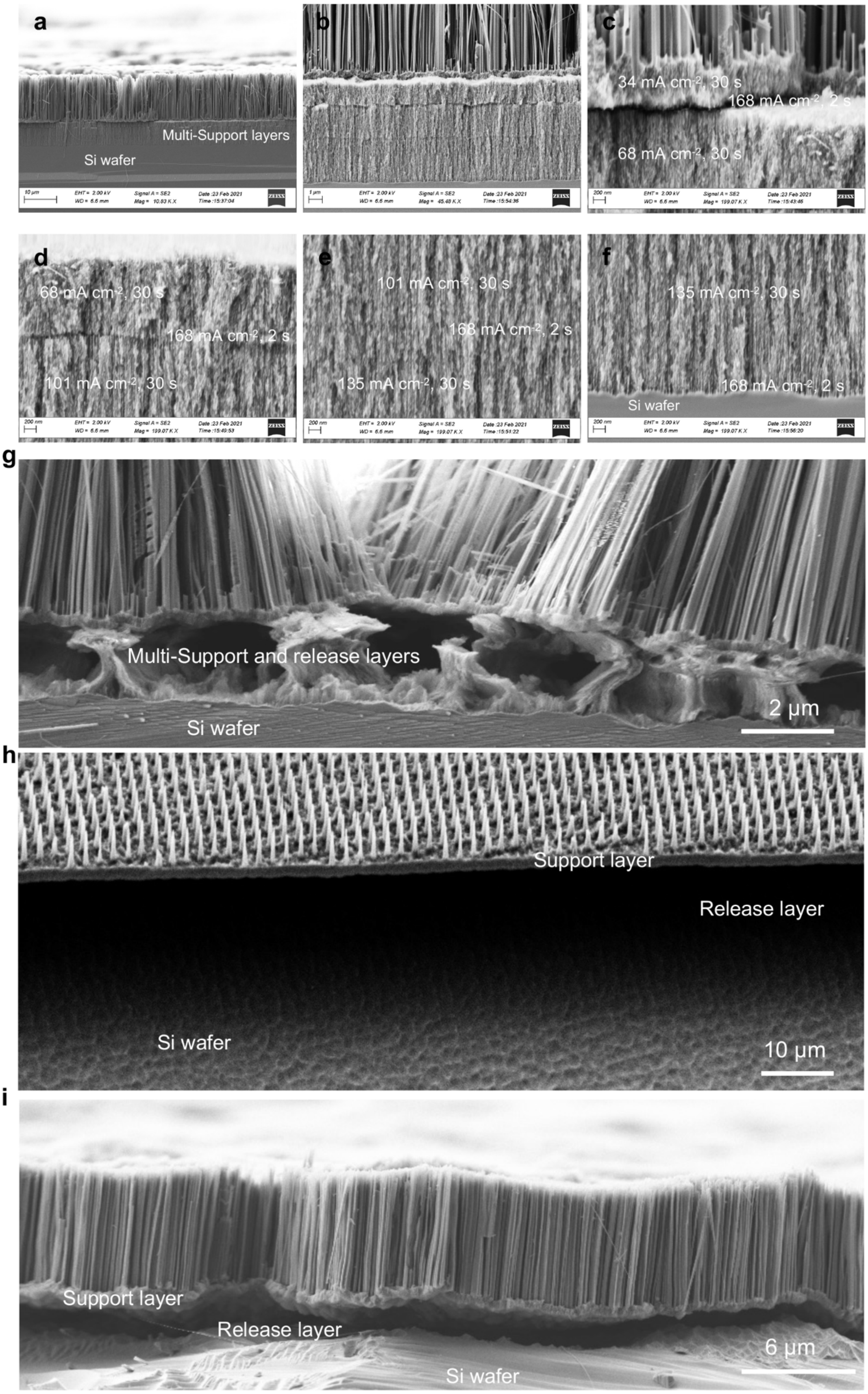

Figure S5. Tilted (45°) SEM micrographs showing (a-f) MACE-generated nanostructures over a supporting multi-layered generated by electrochemical etching with the following etching

parameters: current density: 34 mA cm^{-2}, 30 s, 168 mA cm^{-2}, 2 s; 68 mA cm^{-2}, 30 s, 168 mA cm^{-2}, 2 s; 101 mA cm^{-2}, 30 s, 168 mA cm^{-2}, 2 s; 135 mA cm^{-2}, 30 s, 168 mA cm^{-2}, 2 s; etching solution:1:2 (v/v) electrolyte solution of 50% HF and 99% ethanol. (g-i) Generation of support and release layers. (g) in 1:3 (v/v) mixed electrolyte solution of 50% HF and 99% ethanol, current density: 34 mA cm^{-2}, 30 s, 168 mA cm^{-2}, 2 s; 68 mA cm^{-2}, 30 s, 168 mA cm^{-2}, 2 s; 101 mA cm^{-2}, 30 s, 168 mA cm^{-2}, 2 s; 135 mA cm^{-2}, 30 s, 168 mA cm^{-2}, 2 s; (h) in 1:3 (v/v) mixed electrolyte solution of 50% HF and 99% ethanol at 34 mA cm^{-2}, 120 s, 101 mA cm^{-2}, 6 s. The formation of such high thickness release layer causes the loss of nanoneedles during the washing process. (i) in optimised conditions at 1:3 (v/v) mixed electrolyte solution of 50% HF and 99% ethanol with current density at 34 mA cm^{-2}, 60 s, 101 mA cm^{-2}, 2 s.

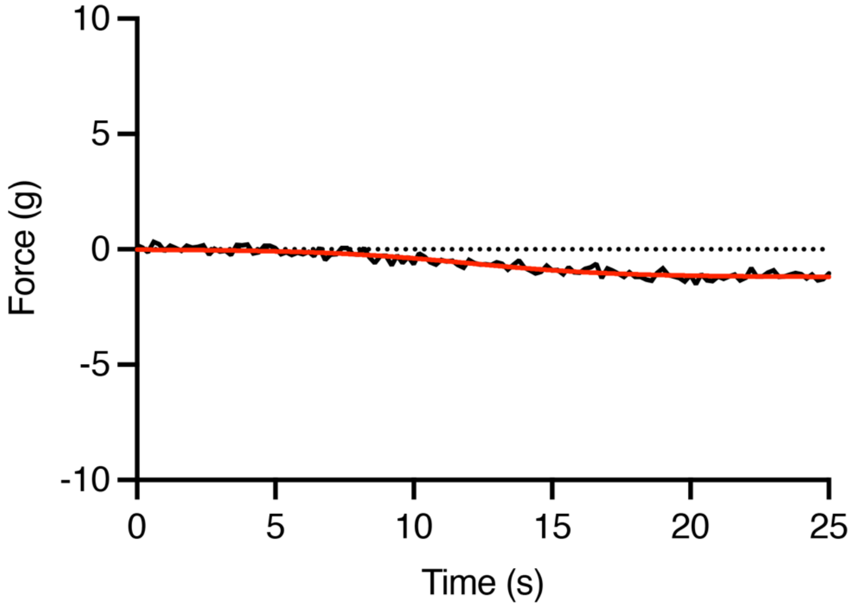

Figure S6. Force measured during the peeling process as a function of time.

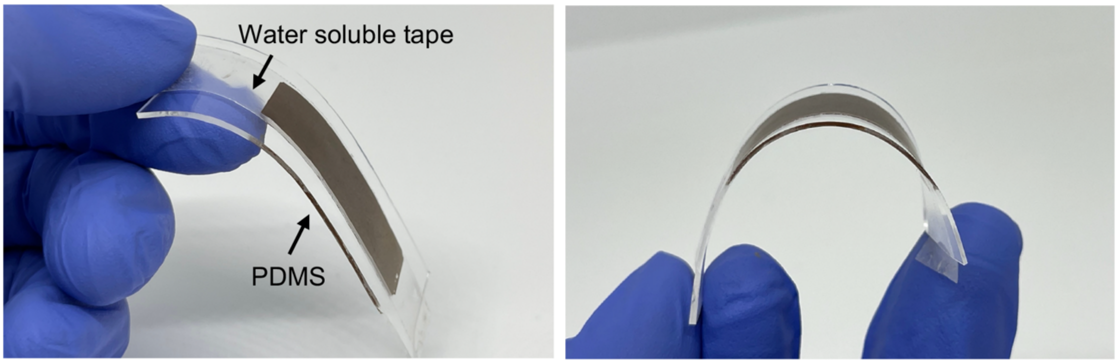

Figure S7. Photographs of the MACE-generated nanostructures carried on the water soluble tape and placed in contact with the receiving substrate (*i.e.*, PDMS).

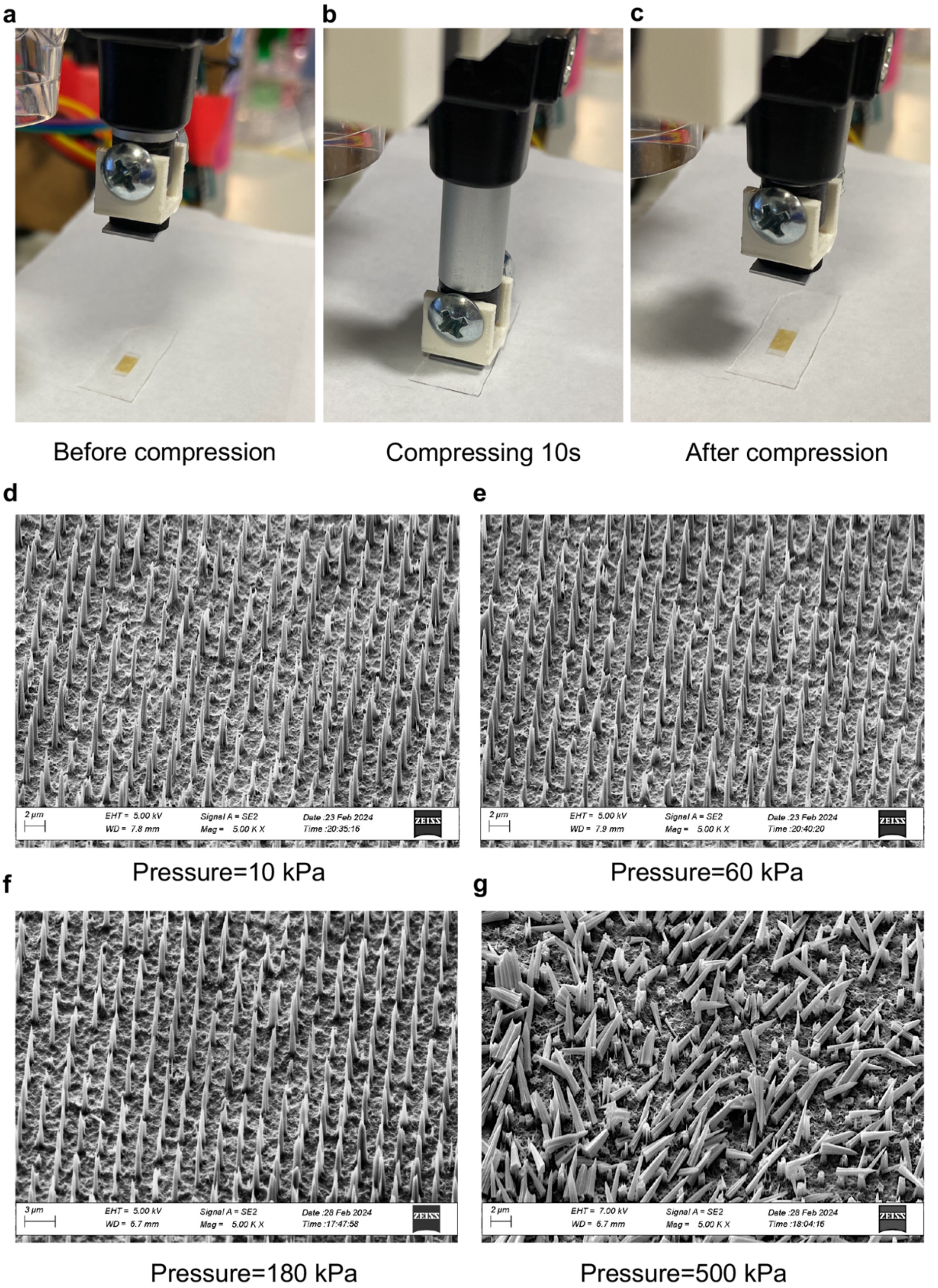

Figure S8. Compression testing of nanoneedles. Experimental set up (a) before compression. (b) during 10 s compression and (c) after compression. (d-g) Tilted (45°) SEM images of nanoneedles subjected to increasing pressure at (d) 10 kPa. (e) 60 kPa. (f) 180 kPa and (g) 500 kPa.

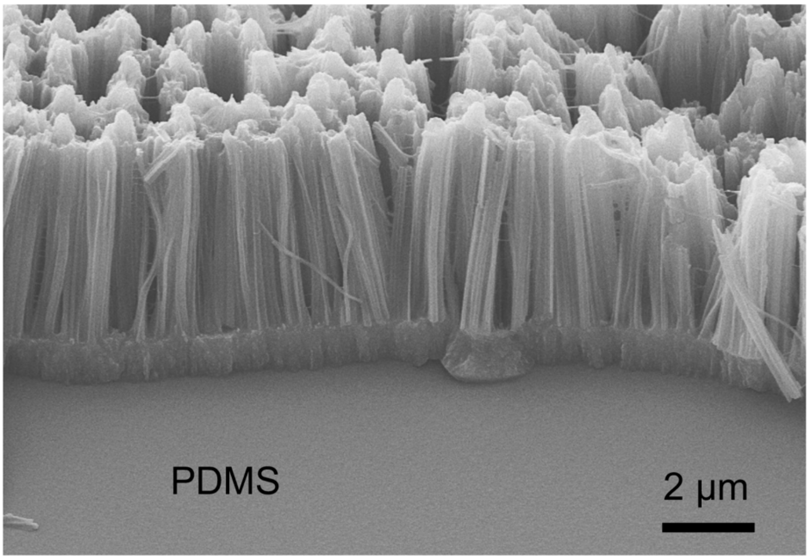

Figure S9. Tilted (45°) SEM image showing the transferred nanopillar arrays with support layer over the receiving substrate.

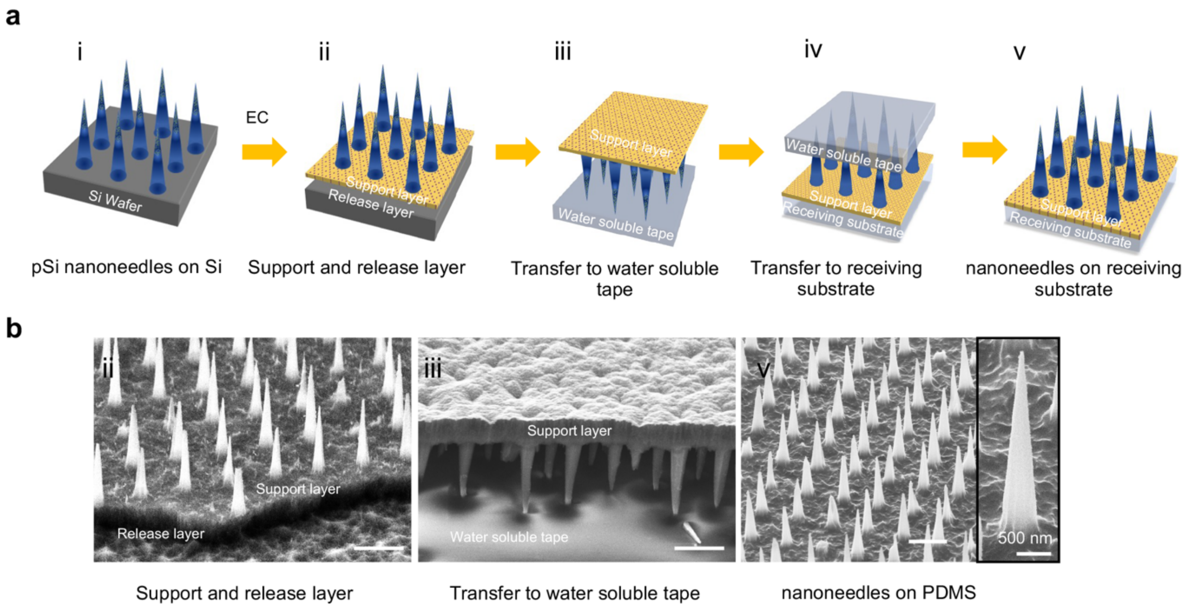

Figure S10. Illustration and SEM characterization of the alternative transfer process, scale bars: 2 µm.

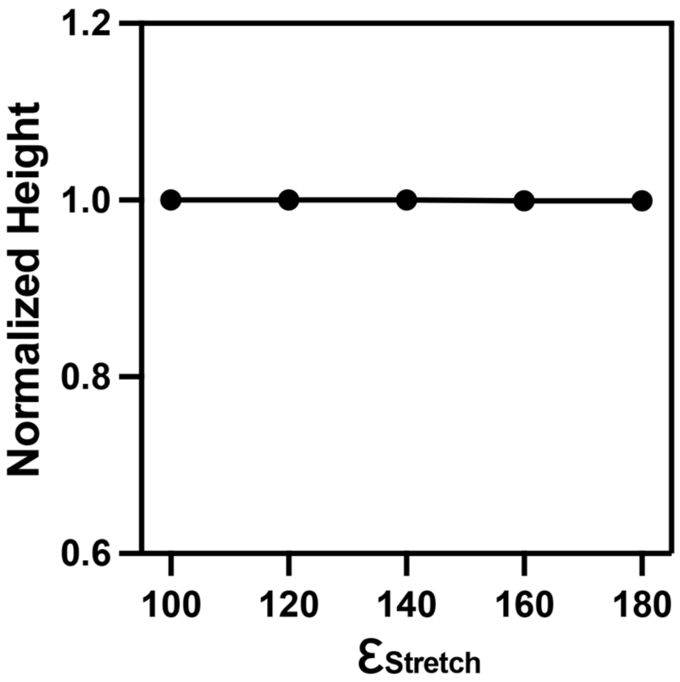

Figure S11. Analysis of nanoneedles height as a function of strain. Normalised to pre-strain height.

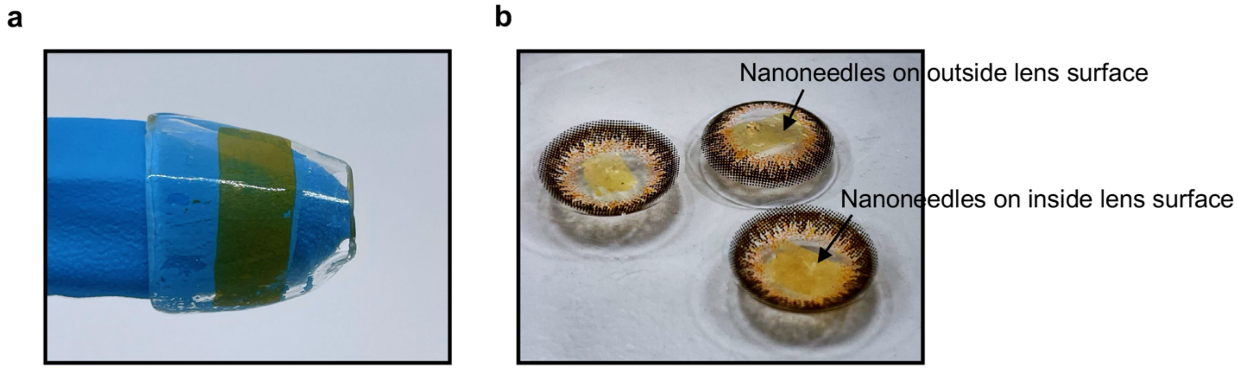

Figure S12. Photographs showing nanoneedles integrated within (a) a flexible gelatin hydrogel and (b) the inner and outer surface of a commercial contact lens (ENVIE).

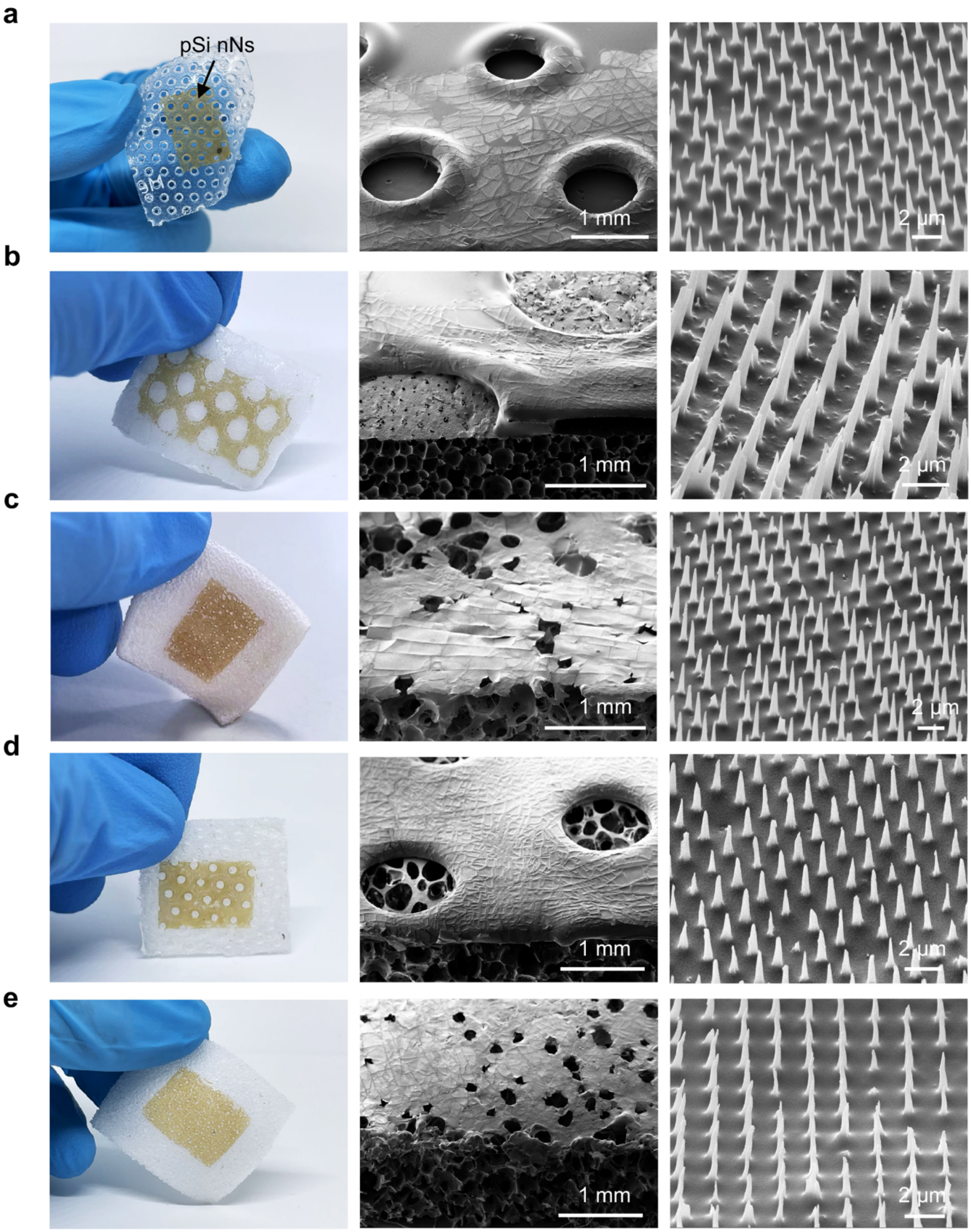

Figure S13. Optical (left) and SEM (middle and right) images of pSi nanoneedles on various wound bandages: (a) Mepilex one. (b) Biatain Silicone lite. (c) Mepilex light. (d) Mepilex border light. (e) Mepilex transfer.

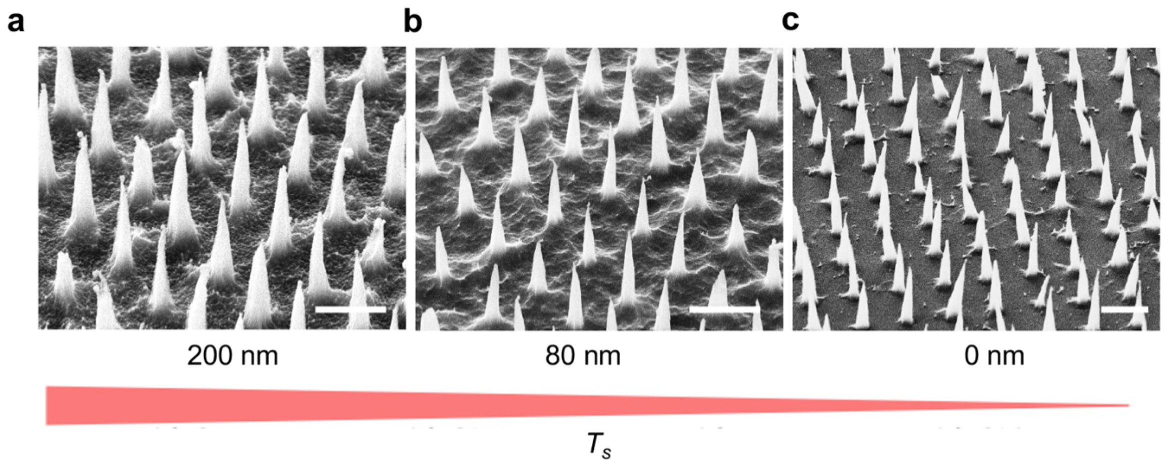

Figure S14. Tilted (45°) SEM image showing the nanoneedles for different T_s: (a) 200 nm. (b) 80 nm and (c) 0 nm. Scale bars, 2 µm.

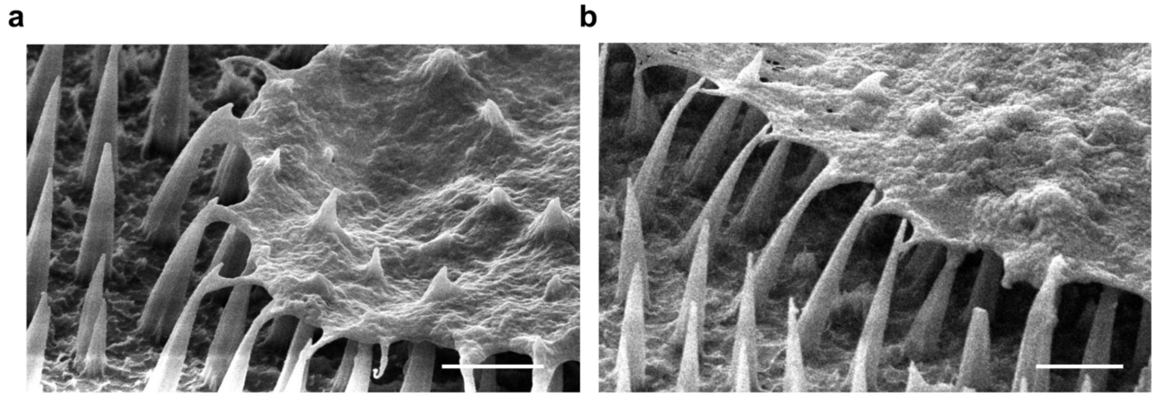

Figure S15. SEM images of hDFs interfacing with nanoneedles with (a) nanoneedles on silicon substrate, (b) nanoneedles on PDMS. Nanoneedles were shown with bending due to cellular forces, scale bars: 2 µm.

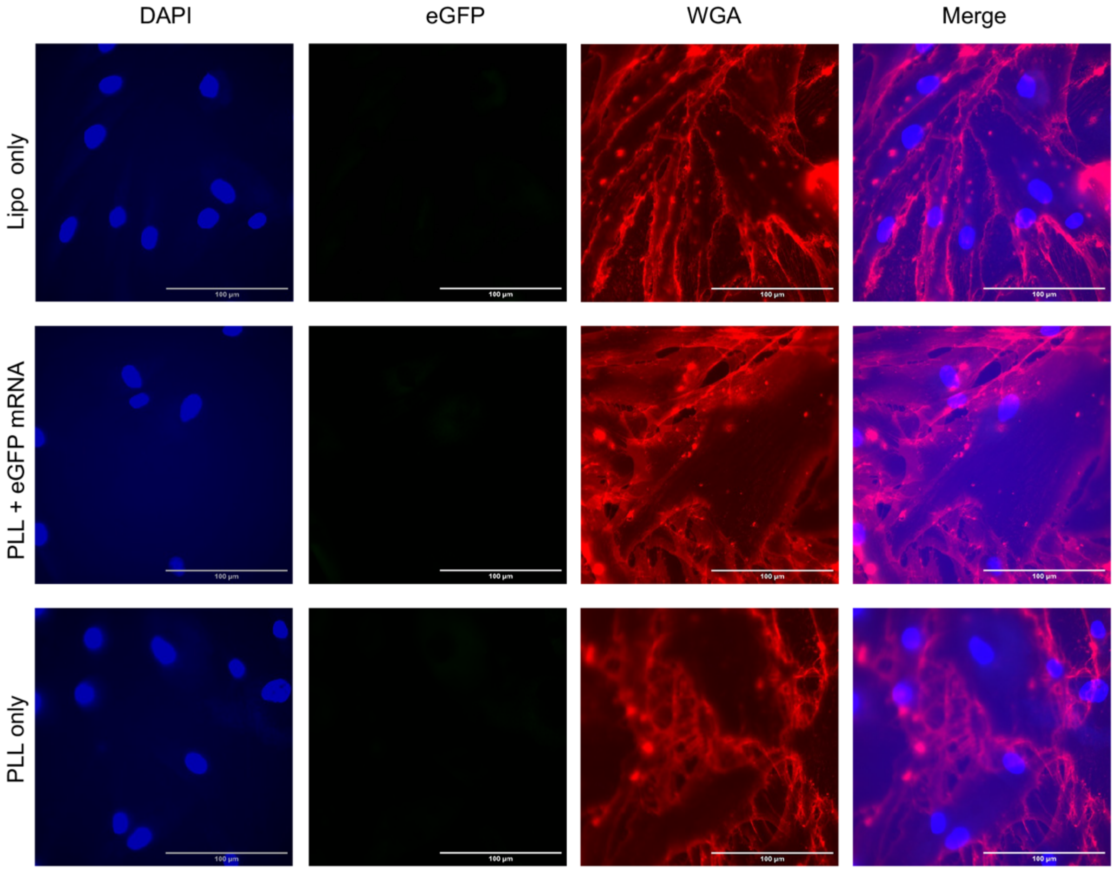

Figure S16. Fluorescence micrsocopy images of control groups for hDF cell GFP mRNA nanoinjection.

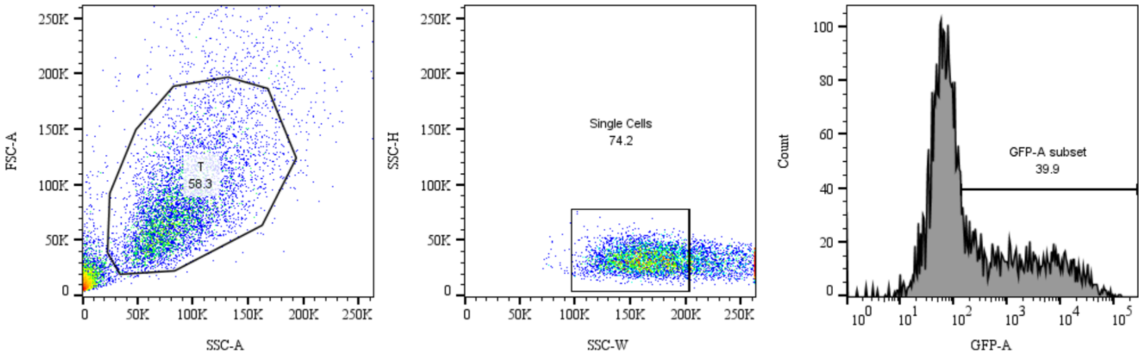

Figure S17. Gating strategy used for flow cytometry analysis– Contour plots and histogram of positive control Lipo samples used for analyzing GFP positive cells.

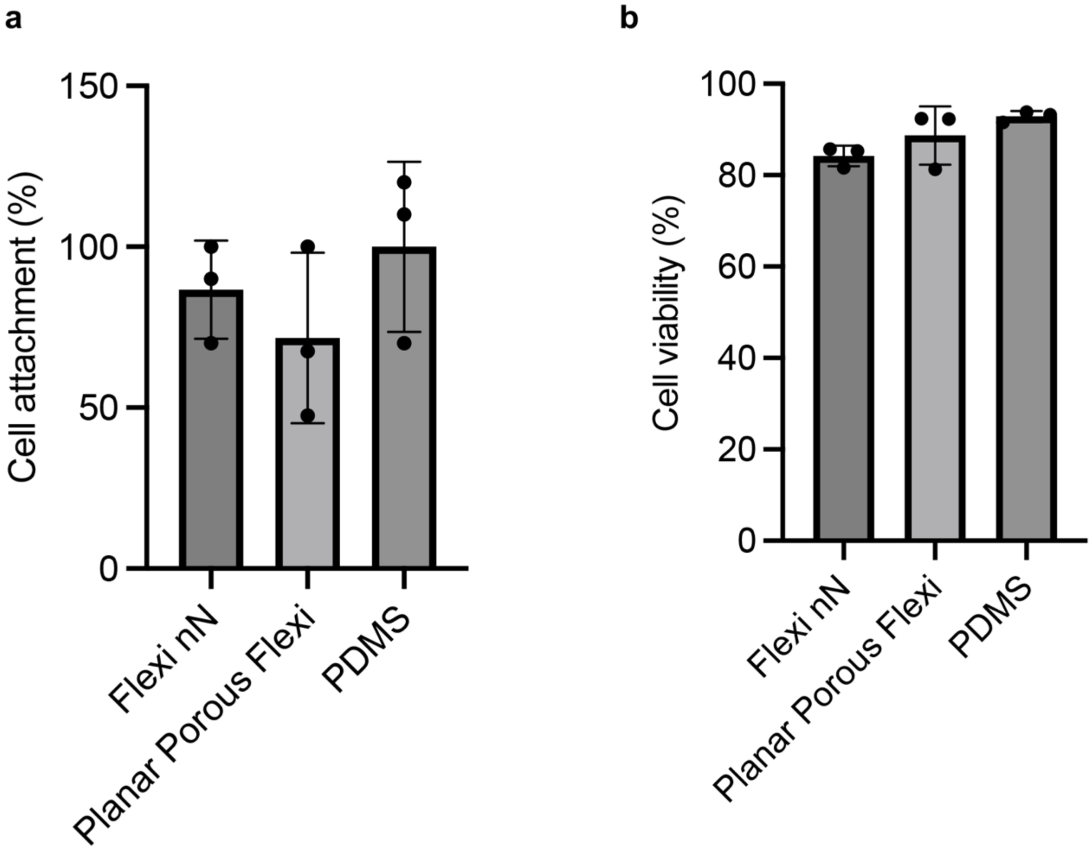

Figure S18. Cell attachment and viability assay (a) Cell attachment on flexible elastomeric substrates. N=3 independent biological replicates. (b) Flow cytometric quantification of cell viability on flexible elastomeric substrates. N=3 independent biological replicates.

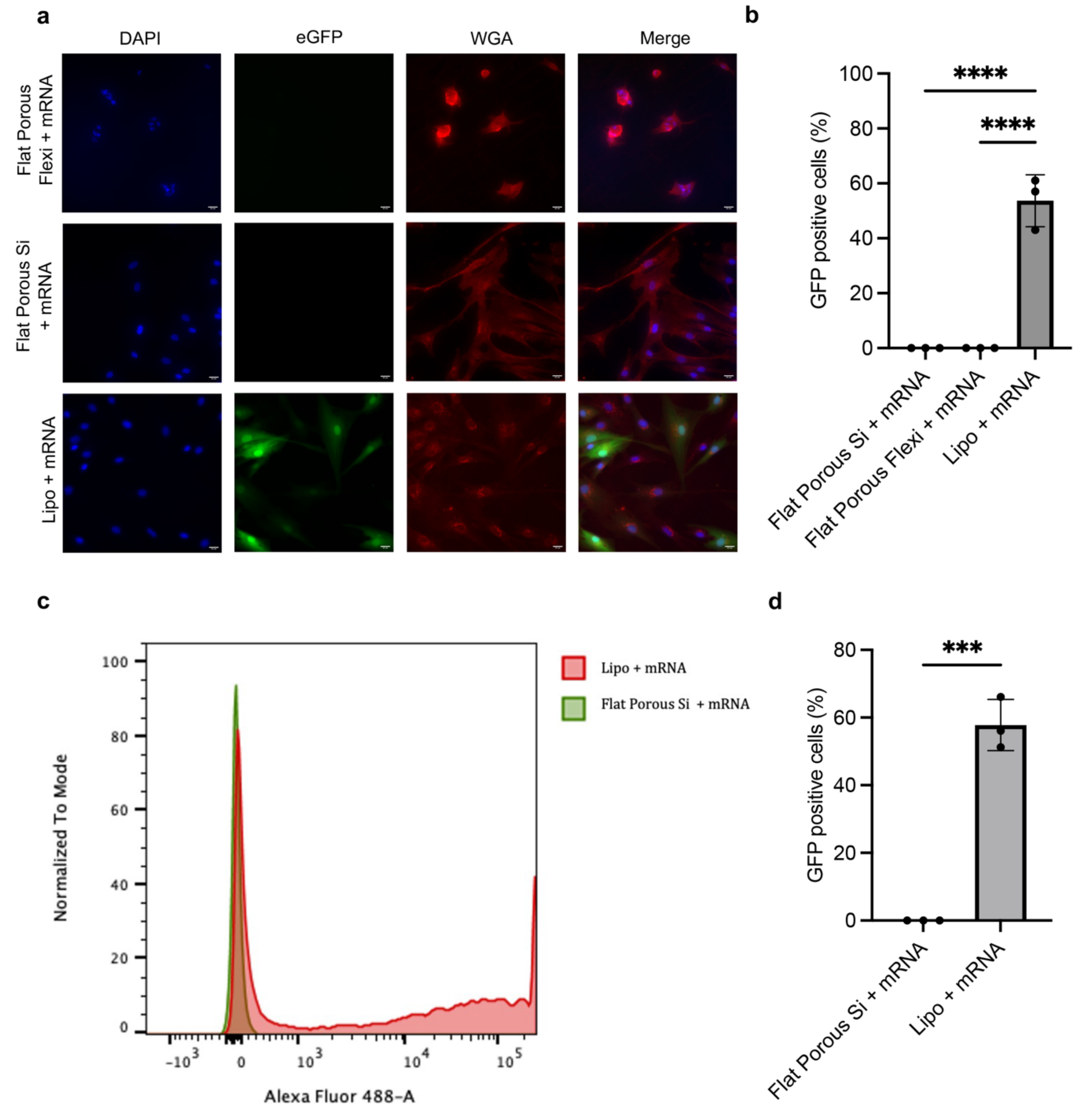

Figure S19. Green fluorescent protein mRNA on planar porous silicon and elastomeric substrates. (a) Fluorescence microscopy images showing negative green fluorescent protein (GFP) expression in primary human dermal fibroblasts (hDFS) when cultured on porous planar silicon and flexible elastomeric substrates. (b) Quantification of the fraction of GFP-expressing cells from the fluorescence microscopy experiment shown in (a). N=3 independent biological replicates, One-way ANOVA with post-hoc Tukey's multiple comparisons test ****p<0.0001. Lipo: Lipofectamine Messenger MAX. (c) Flow cytometric quantification of negative cell fluorescence intensity for cells cultured on planar porous silicon substrate compared to lipofection. (d) Quantification of the fraction of GFP expressing cells from flow cytometry. N=3 independent biological replicates, unpaired t test ***p<0.001.

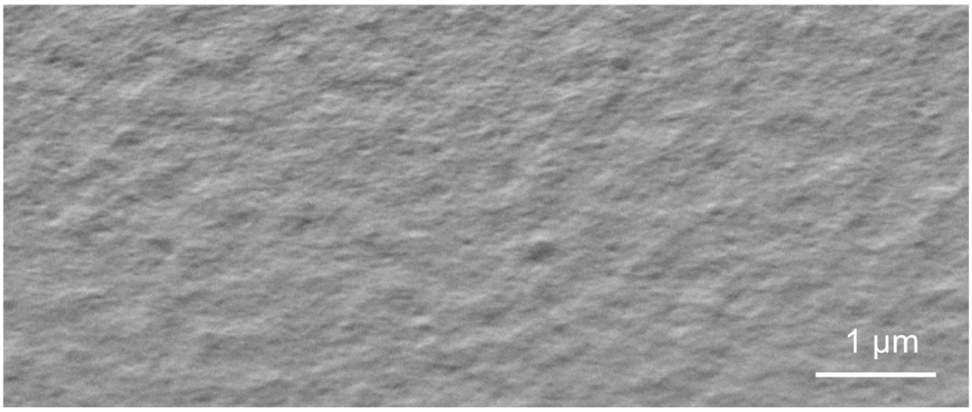

Figure S20. SEM image of the blank agarose surface before the insertion of pSi nanoneedles on elastomeric substrate.

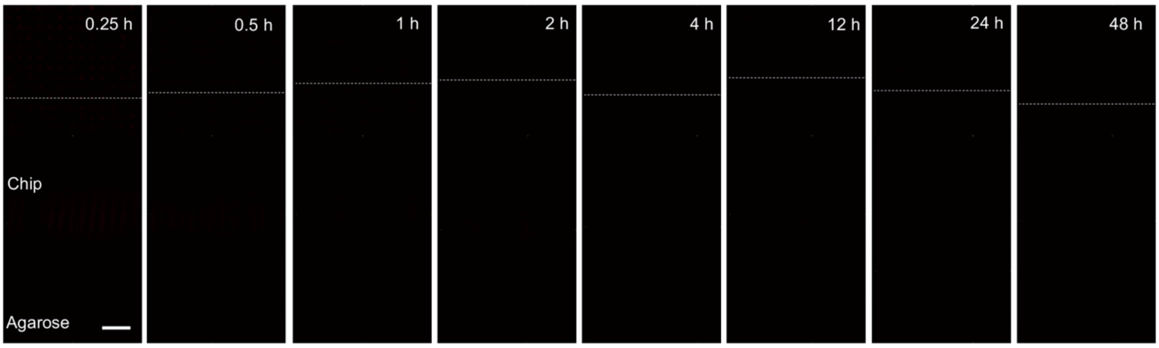

Figure S21. Confocal images of the release profile from a control device without fluorescent payload, scale bars: 5 µm.

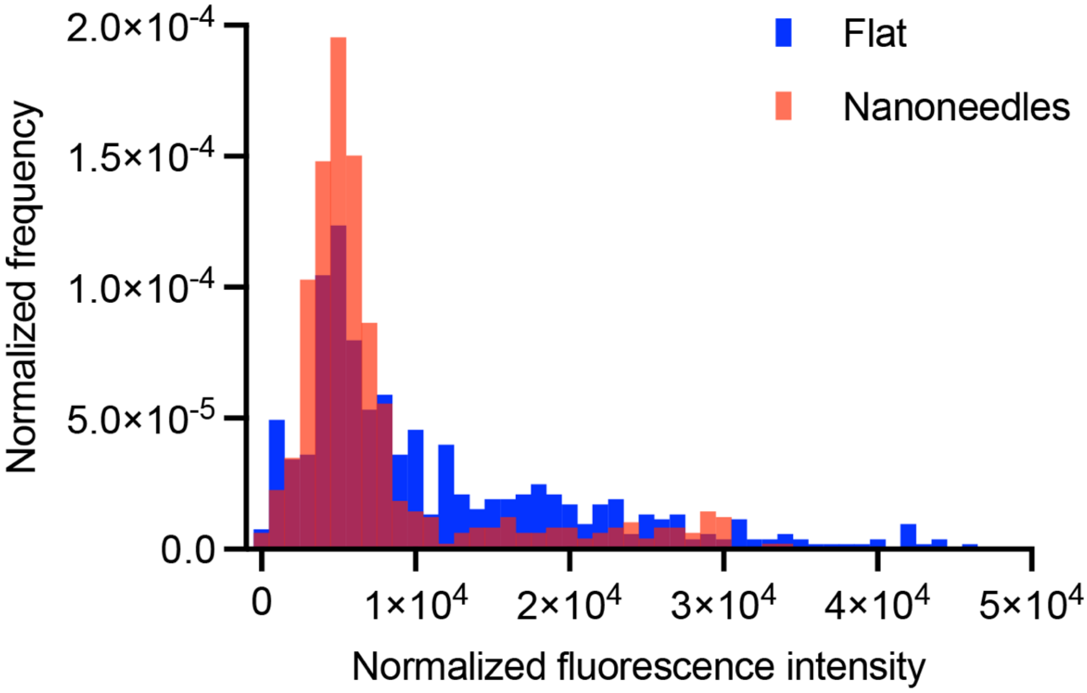

Figure S22. Normalized pixel fluorescence intensity distribution across the 4 independent mouse nanoinjection for flat elastomeric substrates (blue) and nanoneedles (orange).

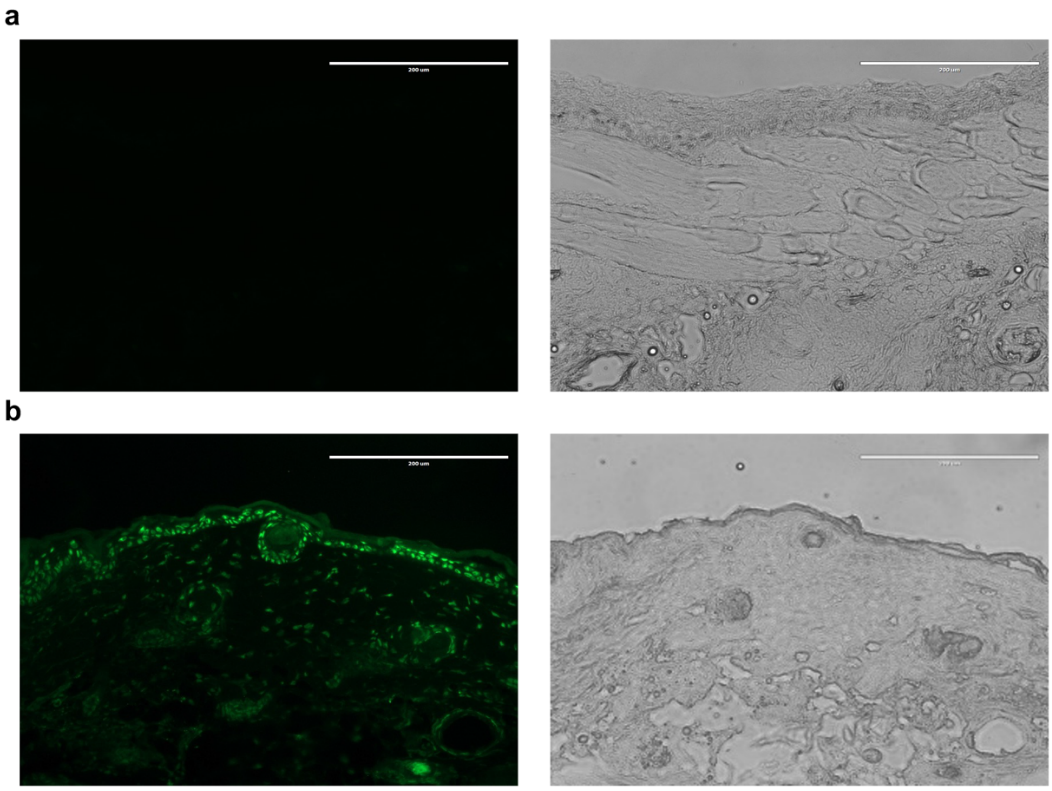

Figure S23. 20X fluorescent TUNEL staining and corresponding bright field images to assess cell death as (a) Nanoneedles on flat elastomer loaded with a fluorescent plasmid. (b) Positive

control. Following fixation and permeabilization, murine skin tissue was treated with Dnase I Recombinant Grade 1 to induce DNA strand breaks.

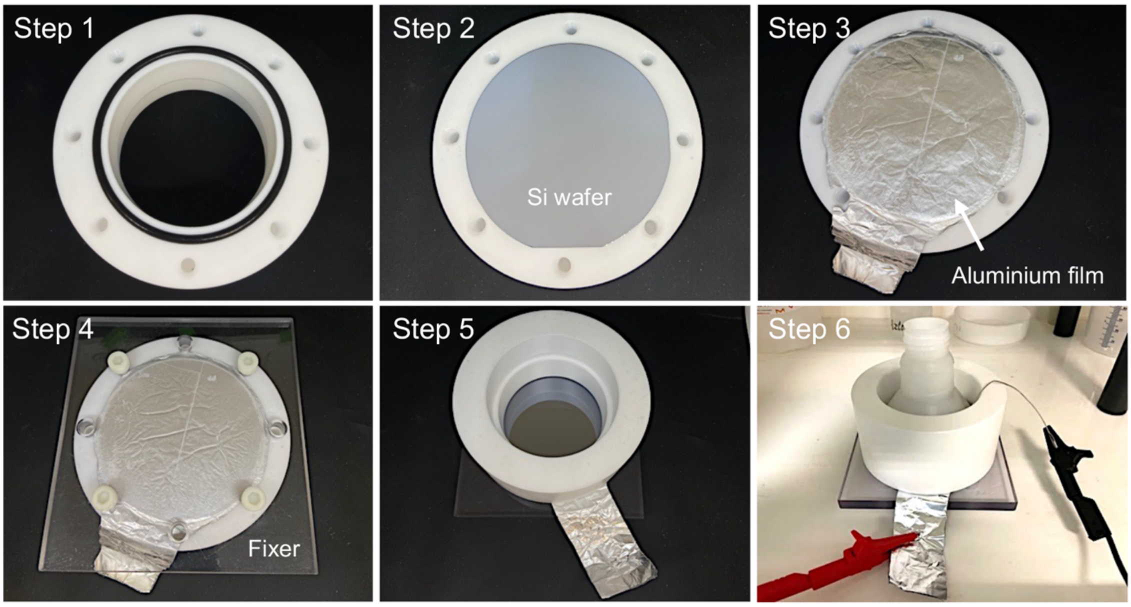

Figure S24. Electrochemical etch setup.

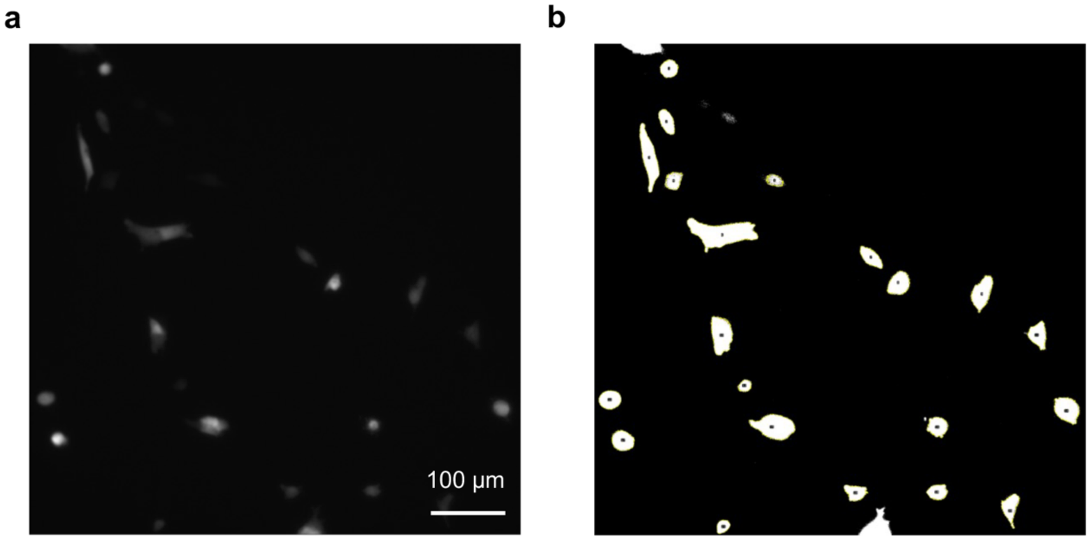

Figure S25. Automated cell segmentation. FIJI macro segmented then labelled and measured cells from each image. (a) Grayscale source data. (b) Segmented and labelled cells of the input image. Results from each image were automatically exported to an excel datasheet. Data cleaning was performed by comparing cells on the original and segmented images and deleting any incorrect segmentation.

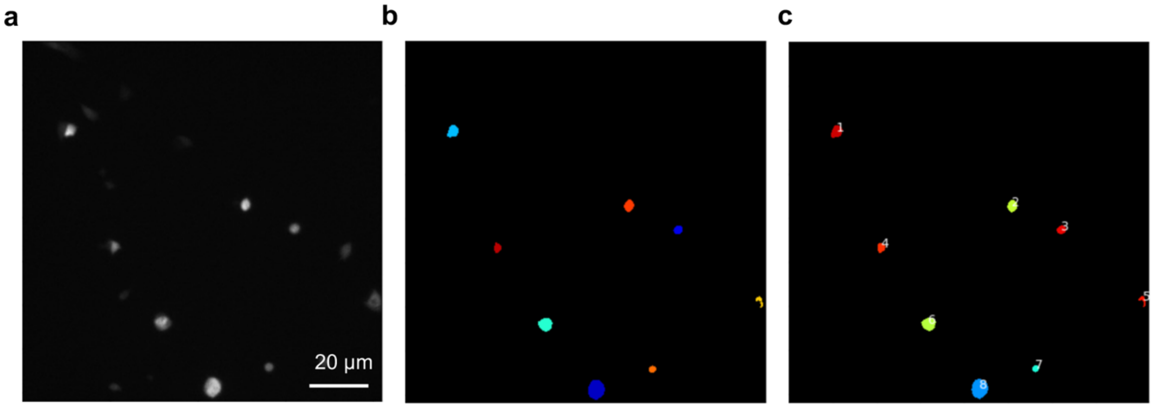

Figure S26. Automated cell tracking. Cell profiler pipeline was implemented to segment and track cells over time. (a) Grayscale source data. (b-c) Segmentation and labeling of the segmented cells from input imaging data. Both X and Y centroid coordinates were recorded for each cell over 36 hours. Coordinates were used to calculate the speed of the cells.
